# Development of a Novel Phagomagnetic-Assisted Isothermal DNA Amplification System for Endpoint Electrochemical Detection of *Listeria monocytogenes*

**DOI:** 10.3390/bios13040464

**Published:** 2023-04-07

**Authors:** Cláudia Maciel, Nádia F. D. Silva, Paula Teixeira, Júlia M. C. S. Magalhães

**Affiliations:** 1 Laboratório Associado, Escola Superior de Biotecnologia, CBQF—Centro de Biotecnologia e Química Fina, Universidade Católica Portuguesa, Rua Diogo Botelho 1327, 4169-005 Porto, Portugal; 2 REQUIMTE/LAQV, Departamento de Engenharia Química, Faculdade de Engenharia, Universidade do Porto, 4200-465 Porto, Portugal

**Keywords:** *Listeria monocytogenes*, bacteriophage P100, magnetic capture, *prfA*, milk analysis, loop-mediated isothermal DNA amplification, electrochemical detection

## Abstract

The hitherto implemented *Listeria monocytogenes* detection techniques are cumbersome or require expensive non-portable instrumentation, hindering their transposition into on-time surveillance systems. The current work proposes a novel integrated system resorting to loop-mediated isothermal amplification (LAMP), assisted by a bacteriophage P100–magnetic platform, coupled to an endpoint electrochemical technique, towards *L. monocytogenes* expeditious detection. Molybdophosphate-based optimization of the bacterial phagomagnetic separation protocol allowed the determination of the optimal parameters for its execution (pH 7, 25 °C, 32 µg of magnetic particles; 60.6% of specific capture efficiency). The novel LAMP method targeting *prfA* was highly specific, accomplishing 100% inclusivity (for 61 *L. monocytogenes* strains) and 100% exclusivity (towards 42 non-target Gram-positive and Gram-negative bacteria). As a proof-of-concept, the developed scheme was successfully validated in pasteurized milk spiked with *L. monocytogenes*. The phagomagnetic-based approach succeeded in the selective bacterial capture and ensuing lysis, triggering *Listeria* DNA leakage, which was efficiently LAMP amplified. Methylene blue-based electrochemical detection of LAMP amplicons was accomplished in 20 min with remarkable analytical sensitivity (1 CFU mL^−1^). Hence, the combined system presented an outstanding performance and robustness, providing a 2.5 h-swift, portable, cost-efficient detection scheme for decentralized on-field application.

## 1. Introduction

*Listeria monocytogenes* is the etiological agent of invasive listeriosis, a severe, albeit sporadic, infectious disease. In 2021, listeriosis was the fifth most reported zoonosis under European Union (EU) surveillance [1], with 96.5% of cases requiring hospitalization and an associated case-fatality rate of 13.7%. This bacterium may occur and efficiently persist in food-processing facilities [2], owing to the complex adaptation mechanisms underlying the remarkable capability to cope with the industrially inflicted sublethal hurdles, challenging pathogen eradication. The long-term persistence and potential post-processing cross-contamination pose a serious food safety concern, being particularly worrisome in ready-to-eat (RTE) foods [3,4,5], which support bacterium growth and are intended for consumption without thermal processing.

The history of gradually evolving listeriosis outbreaks [6] has propelled the introduction of stringent regulatory policies [5,7], rendering compliance with legislation a challenge. Industry commitment to the stipulated policies, particularly the zero-tolerance limit, has triggered the quest for expeditious on-site detection systems to minimize the economic burden of a costly food recall. In fact, the conventional microbiological methods for detecting *L. monocytogenes* in food matrices, although reliable and accurate, are laborious and time-consuming (five to seven days). Hence, the claimed drawbacks of these standard methods provide a compelling argument for the exploitation of rapid approaches [8,9,10], namely nucleic acid amplification-based methods such as PCR (considered the “gold standard” technique) and real-time PCR [11,12]. Nonetheless, these techniques are operationally complex, and cumbersome, requiring non-portable equipment and specialized technicians to perform the analysis. To circumvent these constraints, loop-mediated isothermal amplification (LAMP) [13] has emerged as a valuable on-site nucleic acid amplification procedure owing to the simplicity of the reaction scheme, swiftness, and cost-efficiency. This molecular-based technique accomplishes the target DNA amplification at a single reaction temperature, in an expeditious format, resorting to portable and affordable instrumentation. The high specificity of this method relies on four to six core oligonucleotides which hybridize with six to eight distinct regions on the DNA template. Moreover, LAMP has proven superior analytical sensitivity, vanquishing PCR-based systems performance [14]. Beyond the well-documented outstanding LAMP performance [14,15,16,17], the versatility of the endpoint readout (e.g., turbidimetry, colourimetry, electrochemistry, fluorescence) is an appealing trait, contributing to the practical implementation in resource-scarce laboratories/facilities, or for field purposes.

Noteworthy, LAMP assays may require lengthy pre-treatment procedures to isolate and concentrate the target bacterium from the complex food matrix [18]. These methods are of paramount importance to efficiently cope with matrix interferents and/or inhibitors of the LAMP technique, and concomitantly improve the analytical sensitivity, ergo, lowering the limit of detection. Hence, the quest for rapid pre-analytical concentration approaches has swiftly evolved towards the design of novel bioreceptor-based systems, paving the way for the exploitation of aptamers [14], nucleic acids [10], antibodies [10,19,20], antimicrobial peptides [21], and bacteriophages [22,23]. Amongst these, (bacterio)phages (viruses that specifically infect bacteria) have emerged as auspicious biorecognition elements owing to their remarkable selectivity, sensitivity, and cost-efficient production, also evidencing notable stability to withstand harsh physicochemical conditions.

Notwithstanding the valuable LAMP robustness, the method is devoid of the ability to discriminate between viable virulent cells from non-viable harmless analogues, which may contribute to an overestimation of the bacterial concentration, impairing its implementation for routine monitoring purposes. To address this challenge, complementary procedures have been coupled to LAMP, namely those relying on the DNA-intercalating dye, propidium monoazide (PMA) [20], which specifically diffuses through the damaged cell membrane, hampering the DNA amplification of dead cells. Nevertheless, matrix interferents may compromise the PMA performance, and hence additional pre-processing strategies may be entailed to assure detection reliability. Moreover, this technique depends on the energy-demanding photoactivation of PMA performed with grid electricity, thus hindering the integrated system portability for on-field purposes. This complex procedure precludes the application of an expeditious surveillance system.

Hence, the most promising approach relies on the integration of bacteriophages which, among the quoted traits, possess the unique ability to discriminate the physiological state of the cell. More precisely, phages have been documented to hold great potential in specifically recognizing the viable but non-culturable state (VBNC) [23]. *Listeria monocytogenes* cells may persist in this dormant state in food-processing environments, thereby evading detection using conventional methods. Therefore, phages may constitute a feasible approach to tackle this challenge.

Hitherto, a phagomagnetic-assisted LAMP detection scheme targeting *L. monocytogenes* is still unexplored. In this sense, in the current work, one envisaged the application of the broad lytic spectrum phage Listex™ P100, a member of the *Herelleviridae* family, to propose a novel phagomagnetic separation protocol. This strictly virulent listeriaphage was exploited to selectively pre-concentrate viable cells, and elicit the ensuing bacterial DNA leakage, owing to the occurrence of the host lysis at the last stage of the phage infection cycle. Analogous approaches were formerly documented for the confirmatory identification of *Escherichia coli* using coliphages [24].

The purpose of the present work was to develop a novel all-in-one integrated system comprising a targeted LAMP assay, assisted by a P100–magnetic platform and coupled with an endpoint electrochemical technique, towards a rapid and accurate screening of *L. monocytogenes* along the food chain (farm-to-fork). The analytical performance and applicability of the approach were validated in pasteurized milk, formerly associated with listeriosis outbreaks.

## 2. Materials and Methods

### 2.1. Reagents and Solutions

Magnesium sulfate heptahydrate, Tris hydrochloride, Tween 20 and gelatin from porcine skin, bis(sulfosuccinimidyl)suberate (BS3), and glycerol were purchased from Sigma-Aldrich (St. Louis, MO, USA). Disodium hydrogen phosphate dihydrate and sodium dihydrogen phosphate were acquired from Riedel-de Haën (Seelze, Germany). Sodium hydroxide and sodium molybdate dihydrate were obtained from VWR chemicals (Maia, Portugal). Sodium chloride was purchased from Panreac Quimica S.A (Barcelona, Spain), and methylene blue (MBlue) was acquired from Thermo Scientific (Waltham, MA, USA). All the chemicals were analytical grade or equivalent and used as received without further purification. LISTEX™ P100 bacteriophage was purchased from Micreos Food Safety (Wageningen, The Netherlands). The Micromer^®^-M magnetic particles (Ø, 2 µm) with a magnetite core coated by a styrene-maleic acid-copolymer and with the surface functionalized with PEG-NH_2_ groups (PEG–MBs) were purchased from Micromod^®^ Partikeltechnologie GmbH (Rostock, Germany). Brain heart infusion (BHI) broth and BHI agar were acquired from Biokar Diagnostics (Beauvais, France). The DNA polymerases (*Bst* LF and *Bst* 2.0) and deoxyribonucleotide triphosphates (dNTPs) were obtained from New England Biolabs (Ipswich, Massachusetts, USA). Agarose was purchased from GRiSP Research Solutions (Porto, Portugal). Further information about pH buffer solutions and culture medium preparation is detailed in Section S1 of Supplementary Materials. Ultrapure DNase- and RNase-free water were used for DNA amplification experiments.

### 2.2. Microorganisms and Inoculum Preparation

#### 2.2.1. Bacterial Strains and Culture Conditions

*Listeria monocytogenes* EGD-e (ATCC BAA-679) (phage P100 susceptible) was used as the reference strain to develop and evaluate the phagomagnetic-assisted LAMP procedure and to perform downstream applications. A cohort of *L. monocytogenes* and *Listeria* spp. strains detailed in Table 1 were comprehensively selected to evaluate the LAMP system performance.

Stock cultures were preserved at −80 °C in BHI broth supplemented with 20% (*v*/*v*) glycerol. Prior to each experiment, the bacterial strains were routinely streaked onto BHI agar and incubated overnight at 37 °C. Afterwards, a single colony was inoculated into BHI broth and grown at 37 °C until the late exponential phase, and sub-cultured (1%, *v*/*v*) onto fresh medium, under the indicated growth conditions.

#### 2.2.2. Bacteriophage Titration by the Double-Layer Method

The phage Listex™ P100 stock solution presented an initial titre of 10^11^ plaque forming units (PFU) mL^−1^ and was stored in the original saline buffer at 4 °C.

The phage titration was performed according to the double-layer method (plaque assay), as formerly described by Kropinski et al. [25]. Briefly, phage samples (MB-immobilized or in their free form) were serially 10-fold diluted in SM buffer. The host culture (100 μL of overnight grown *L. monocytogenes* ATCC 19116) and aliquots of 100 μL of the decimal phage dilution were mixed with 3 mL of molten LC soft agar. The suspension was poured onto BHI agar plates and incubated at 30 °C. Plaque forming units enumeration was performed 24h post-infection.

### 2.3. Preparation of P100 Modified Magnetic Particles

Commercial PEG–MBs (20 μL, 10 mg mL^−1^ in H_2_O) were used as a magnetic platform for P100 bacteriophage loading. P100 was physically immobilized resorting to an optimized three-step protocol [26] comprised of: (i) PEG–MBs sterilization (ethanol 97%, 30 min); (ii) bacteriophage immobilization (140 μL of 1 × 10^9^ PFU mL^−1^ of P100, in 0.01 M citrate buffer pH 5 or only citrate buffer in blank assays, incubated overnight at 350 rpm, 4 °C) and; (iii) microparticle active binding sites blockage (bovine serum albumin (BSA) 1% (*w*/*v*) in 0.1 M PBS pH 7.4, for 8 h, at 350 rpm, 4 °C); all interspersed by several washing steps (3× with 0.01 M PBST after step (i), 2× with 0.01 M PBS after steps (ii), and 1× after (iii)). In the end, the separated bacteriophage-functionalized magnetic particles (P100–MBs) were resuspended in 500 μL of SM buffer (particle concentration of 0.4 mg mL^−1^). All samples were stored at 4 °C until used. A PCMT Thermoshaker from Grant Instruments (Shepreth, UK) was used for all incubation steps with temperature control. A MagJET separation rack from Thermo Scientific™ was used to perform magnetic separation between the incubation and washing steps.

The effect of the immobilization method (physical or chemical) was also evaluated through the application of a covalent immobilization protocol [26], using the bis(sulfosuccinimidyl)suberate (BS3) crosslink between amine surface groups of PEG–MBs and surface amine moieties of P100 particles. Briefly, 10 mM of BS3 in 0.01 M PBS was added to the sterilized PEG–MBs suspension and allowed to react under orbital shaking (350 rpm) for 30 min. Afterwards, the particles were washed three times with PBST and incubated with P100 phage particles (10^9^ PFU mL^−1^, 0.01 M PBS) overnight at 4 °C using the thermo-shaker.

The titre of non-immobilized P100 phages in the supernatant (*PFU supernatant*) and the initial titre (*PFU initial*) were determined by the double-layer method. The immobilization efficiency (*IE*) was calculated following Equation (1).

(1) $$IE\left(\%\right)=\left(1-\frac{PFU\;supernatant}{PFU\;initial}\right)\times 100$$

### 2.4. Phagomagnetic Separation Protocol: Capture Efficiency

A stationary-phase culture of *L. monocytogenes* EGD-e was 100-fold diluted in BHI and grown at 37 °C to exponential phase (optical density at 600 nm (OD_600 nm_) equal to 0.6). The cells were then harvested by centrifugation (4000× *g*, for 10 min, at room temperature) and washed thrice with SM buffer. To ascertain the colony-forming units (CFU) of the initial inoculum, the obtained bacterial cell suspension was 10-fold serially diluted in PBS and plated onto BHI agar.

Afterwards, for magnetic separation and pre-enrichment, 500 μL of the *L. monocytogenes* suspension (10^3^ CFU mL^−1^) were added into P100-modified magnetic particles (P100–MBs) previously washed twice with 0.1 M Tris pH 7.2.

**Figure 1:**
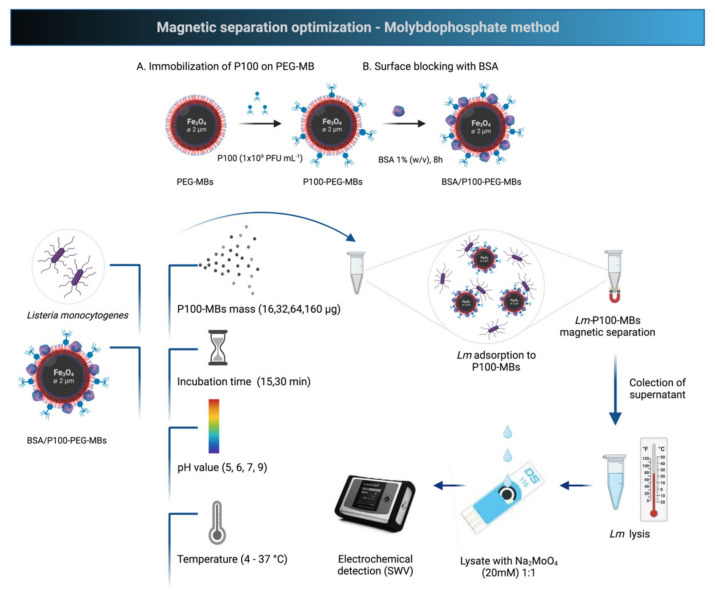
Schematic illustration of the optimization of the phagomagnetic separation protocol resorting to the molybdophosphate method.

The optimization of the magnetic separation protocol (depicted in Figure 1) was performed using the molybdate assay procedure [27]. Briefly, the *L. monocytogenes* cells were incubated with the P100–MBs under orbital shaking (250 rpm, 25 °C) for 15 and 30 min. Subsequently, P100–MBs probes with captured cells (*Lm*-P100–MBs) were magnetically attracted, and the supernatant was exposed to 90 °C for 10 min to accomplish the bacterial cells’ thermal lysis. Afterwards, the lysate suspension was mixed 1:1 with Na_2_MoO_4_ (20 mM) on a screen-printed carbon working electrode (DRP-110, from DropSens, Oviedo, Spain) for 15 min before electrochemical measurements. All the results were correlated with a calibration plot (*L. monocytogenes* cells in CFU mL^−1^ against molybdate peak current intensity) performed daily, under the same experimental conditions. Square wave voltammetry (SWV) scans were obtained with a potentiostat/galvanostat PalmSens 4 (Houten, The Netherlands), using 0.02 V of amplitude and a frequency of 5 Hz. Capture efficiency (%) and specific capture (%) were calculated using Equations (2) and (3), respectively.

(2) $$Capture\;efficiency\;\left(\%\right)=\frac{Lm\;cell\;number\left(initial\right)-Lm\;cell\;\;number\;\left(supernatant\right)\;\;}{Lm\;cell\;number\left(initial\right)}\times 100$$

(3) Specific capture=% capture with P100MBs−% capture with blankMBs

The effect of P100–MBs mass (16, 32, 64, and 160 µg), pH (5, 6, 7, and 9), and temperature (4, 11, 25, and 37 °C) on capture efficiency and specific capture were studied following this protocol.

### 2.5. Development of a Novel LAMP Assay Targeting prfA

#### 2.5.1. Preparation of Genomic DNA

The extraction of the bacterial genomic DNA was performed with a commercial genomic DNA extraction kit (GRS Genomic DNA Kit Bacteria, from GRiSP Research Solutions) according to the manufacturer’s instructions. The DNA concentration was estimated utilizing Qubit™ 1X dsDNA High Sensitivity Assay Kit and the respective fluorometer (Invitrogen, Waltham, MA, USA), and the corresponding DNA integrity was evaluated using agarose gel (0.8%, *w*/*v*) electrophoresis. The DNA quality was assessed with the NanoDrop™ One Spectrophotometer (Thermo Scientific).

#### 2.5.2. Design of the *L. monocytogenes*-Specific LAMP Primers

The positive regulatory factor A (PrfA)-encoding gene was selected as the specific target for the *L. monocytogenes* detection. The sequences of the pleiotropic regulatory gene were retrieved from the National Center for Biotechnology Information database. Afterwards, ClustalW sequences alignment was performed to identify the conserved regions. Three sets of primers targeting the consensus *prfA* sequence were generated (Table S1 in Supplementary Materials) using Primer Explorer software. Each primer set comprised four core primers, recognizing six distinct regions of the DNA template, namely two outer displacement primers, F3 and B3 (forward and backward outer primers), and two inner primers, FIP and BIP (forward and backward inner primers, designed with the intent to hybridize with the complementary and reverse complementary target sequences, respectively).

The melting temperature value of the oligonucleotide sets was determined theoretically. The corresponding GC content, secondary structure formation (hairpin structures), and dimerization were evaluated in silico using the OligoEvaluator software. The specificity of the designed primers was examined in silico utilizing the Basic Local Alignment Search Tool to guarantee that the selected oligonucleotides were unique to the desired target sequence and that probe efficiency was not negatively impacted owing to off-target interactions.

The DNA oligo primers utilized for LAMP amplification were synthesized (high-performance liquid chromatography (HPLC) purified) by Stab Vida (Caparica, Portugal) and are listed in Table S1 in the Supplementary Materials.

#### 2.5.3. Optimization of LAMP Reaction System

Optimization of the LAMP reaction conditions comprised the evaluation of different concentrations of the following components: deoxyribonucleotide triphosphates (dNTPs; 0.2 to 1.4 mM), magnesium sulphate (MgSO_4_; 0.5 to 4 mM), along with the determination of the optimum primers ratio (1:1−1:10). The *Bst* LF and *Bst* 2.0 DNA polymerase activity performance was also compared. The optimal reaction system consisted of 0.2 μM F3/B3 primers, 0.8 μM FIP/BIP, *Bst* 2.0 polymerase (8 U), 0.3 mM dNTPs, 2 mM MgSO_4_, 20 mM Tris–HCl, 10 mM (NH_4_)_2_SO_4_, 50 mM KCl, 0.1% (*v*/*v*) Tween 20 (pH 8.8). In each assay, nuclease-free water was included as a negative control. The LAMP amplification was conducted in a heating block at 62 °C for 50 min and terminated following a heat inactivation at 80 °C for 5 min. LAMP amplicons were resolved on 2% (*w*/*v*) agarose gel.

#### 2.5.4. Evaluation of LAMP Assay Specificity

The inclusivity of the newly developed LAMP method was evaluated by resorting to a cohort of 61 *L. monocytogenes* strains (Table 1), comprising representatives of the most relevant serotypes (1/2a, 1/2b, 1/2c, and 4b). In line with the proposed food-focused application of the assay, strains isolated from dairy specimens were also included in this cohort.

The potential cross-reactivity (exclusivity) of the LAMP technique was further investigated. A panel consisting of genomic DNA samples from Gram-positive and Gram-negative bacteria (Table 1) was analysed using the novel assay system. This cohort ranged from closely related *Listeria* species to distantly related species, including competitive microbiota (particularly those prevailing in pasteurized milk, namely mesophilic bacteria), with a focus on a dairy-related application.

#### 2.5.5. Determination of Analytical Sensitivity

The LOD_95_ is defined as the concentration of the target DNA at which an amplicon is detected with a probability of 0.95, being estimated by probit regression analysis. For this purpose, genomic DNA from an overnight culture of *L. monocytogenes* EGD-e was isolated and fluorometrically quantified on Qubit. The bacterium genome is 2.9 Mb long, harbouring a single copy of *prfA.* The corresponding copy number of the gene was determined based on the molecular weight of the double-stranded DNA template, following Equation (4) formerly described [28].

(4) $$Copies\;of\;template={\frac{ng\;of\;double\;stranded\;DNA\times Avogadro’s\;constant\;}{Length\;in\;base\;pairs\times 10^{9\;}\times 650\;Da}}^{}$$

Experiments were performed on 10-fold serially diluted genomic DNA ranging from 39 ng μL^−1^ to 0.39 fg μL^−1^ to determine the limit of detection (LOD_95_). These DNA concentration values corresponded to 1.25 × 10^7^ and 0.125 copies, respectively, of the genome per LAMP reaction.

#### 2.5.6. PCR Targeting *prfA*

A formerly developed PCR procedure [29,30] targeting *prfA* of *L. monocytogenes* was used as a standard for comparison with the novel LAMP assay. The master mix comprised 3 mM of MgCl_2_, 150 mM dNTPs, 0.25 μM of each primer (LIP1 and LIP2, Table S1), 1 U Taq DNA Polymerase, 1× Taq Buffer (Thermo Fisher Scientific) and 0.8 ng of DNA. The amplification was performed in a T100 thermal cycler (Bio-Rad Laboratories, Hercules, CA, USA) and PCR products were resolved on 0.8% (*w*/*v*) gel electrophoresis.

### 2.6. Validation of the Applicability of the Developed Method in Pasteurized Milk

#### 2.6.1. Application of Phagomagnetic-Assisted LAMP Method in Milk Samples

**Figure 2:**
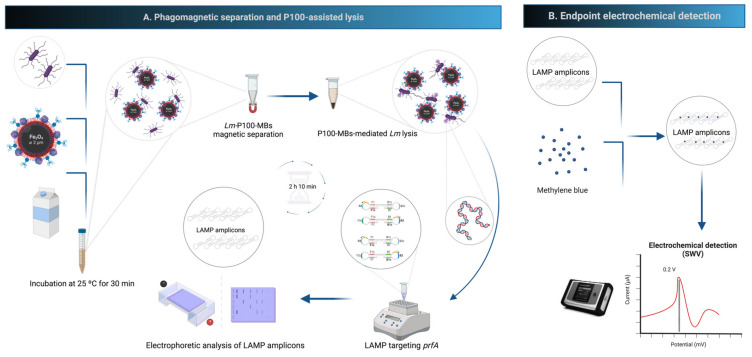
Schematic illustration of the validation of the integrated detection system.

The proof-of-concept of the optimized combined system for the rapid and accurate detection of *L. monocytogenes* was performed in whole pasteurized milk (depicted in Figure 2A). This food product was selected as a representative model of dairy matrices, which are often associated with listeriosis outbreaks.

Pasteurized whole milk was purchased from a local supermarket (Porto, Portugal) and confirmed for the absence of culturable *L. monocytogenes* cells according to the ISO 11290-1:2017 standard [31]. Afterwards, 25 mL of pasteurized milk were aseptically transferred to sterile Falcon tubes, spiked with decimally diluted *L. monocytogenes* cell suspensions to obtain bacterial loads in the range of 10 to 10^3^ CFU mL^−1^, and thoroughly homogenized. The *Listeria* inoculum volume represented 1% of the total sample volume [32]. In parallel, for each challenging experiment, an un-spiked milk sample was used as a negative control. Three independent replicates were prepared for each sample. The previously optimized magnetic separation protocol (Section 2.4) was performed to capture and pre-concentrate bacterial cells from those artificially contaminated samples. In brief, 10 mL of the spiked and un-spiked samples were divided into 1 mL aliquots, and 64 µg of P100–MBs were added to each aliquot, and the mixture was incubated at 25 °C under orbital shaking for 30 min. The same protocol was also conducted with magnetic particles devoid of P100 (negative control).

The magnetic separation procedure was performed, and the complex *Lm*-P100–MB from each aliquot was resuspended in PBS, pooled, and spread-plated onto BHI and PALCAM *Listeria* selective agar for enumeration of adsorbed *L. monocytogenes* cells. Four independent experiments were performed in triplicate. Concomitantly, *L. monocytogenes* suspensions with theoretical 10 or 50 cells per 10 mL were prepared from the 10^2^ CFU mL^−1^ bacterial cell suspension and submitted to the above detailed magnetic separation protocol.

The collected *Lm*-P100–MB complex was rinsed thrice with PBS, resuspended in BHI, and incubated at 30 °C, for an additional period of 30 min to elicit the lytic cycle (total incubation time of 60 min). Afterwards, the lysed samples were centrifuged at 12,000× *g* for 5 min, the supernatant was collected, and the released genomic DNA was LAMP amplified. These experiments included the target sample, a blank (*Lm*–*MB*) control, as well as a positive control (DNA extracted from *L. monocytogenes* pure culture), and a negative control (nuclease-free water). The amplicons, and corresponding control samples, were resolved by conventional gel electrophoresis. The LOD_95_ of the P100–MB-assisted LAMP method was determined using a logistic regression model.

#### 2.6.2. Endpoint Electrochemical Detection

Methylene blue was used as an intercalator redox probe, and a reduction of the MBlue peak current intensity was observed in the presence of the LAMP amplification products (Figure S8 in the Supplementary Materials). The LAMP product was mixed 1:1 with methylene blue solution and left to react for 15 min. Afterwards, 20 µL of the mixture was dropped on a screen-printed carbon electrode (AC1-W4-R2, from BVT Technologies, Strázek, Czech Republic), and the square wave voltammetry (SWV) scans were obtained with a portable potentiostat/galvanostat from PalmSens 4, using 0.025 V of amplitude, potential step 0.004 V and a frequency of 100 Hz (as depicted in Figure 2B). Various MBlue concentrations (5–25 mM) were investigated to determine the suitable amount of the redox probe.

### 2.7. Statistical Analysis

Statistical analysis was performed using SPSS Statistics software version 28 (IBM^®^, Chicago, IL, USA). Analysis of variance (ANOVA) was used to determine differences between groups (with Tukey´s post hoc test for pairwise comparisons) when all assumptions necessary were validated, namely normality and homoscedasticity of data. Normality was assessed by Shapiro-Wilk or Kolmogorov-Smirnov tests and homogeneity of variances by Levene’s test. Non-parametric alternative tests were used when these assumptions were not verified, namely Kruskal-Wallis and Mann-Whitney tests, respectively. The significance level assumed in all tests performed was 5%.

## 3. Results and Discussion

### 3.1. Effect of Incubation Solution pH and Immobilization Method

Virions, such as phage P100 are permanent dipoles, with a negatively charged head and positively charged tail, being able to orient their capsids or tails on a charged surface due to electrostatic interactions [33,34]. Hence, the determination of the surface charge of PEG-NH_2_–MBs and P100 is a requisite for the proper characterization of MBs and indispensable for the development of physisorption immobilization protocols [35]. The isoelectric point of PEG-NH_2_–MBs particles was estimated to be approximately 5.3 (Figure S1a in Supplementary Materials). Therefore, it is expected a positive surface net charge below pH 5.3 and a negative charge at higher pH values. P100 Dynamic Light Scattering (DLS) experiments (Figure S1b in Supplementary Materials) suggest aggregation or changes in the phage configuration at pH ≤ 4.4, according to the expected P100 size of about 90 × 198 nm (head × tail) [36]. These results indicate that aggregation is not dependent on the isoelectric point of the whole phage P100 [37], previously predicted at 5.67 [38], reflecting the individual amino acid composition of their capsid/tail [39]. Hereupon, to avoid conflicting effects of virus aggregation, the optimization of immobilization protocol proceeded within a pH range of 4 to 9 in which phages were preferentially monodispersed.

The immobilization of phage particles on the surface of the PEG-NH_2_–MBs does not guarantee successful bacterial capture nor the antibacterial lytic activity of the functionalized magnetic material [40]. Listex P100 is a tailed, net-charged asymmetric phage, capable to recognize and adsorb to *L. monocytogenes* by phage receptor-binding proteins present in the tail [41]. Thus, the proper orientation of the immobilized phage on the magnetic particle is a key point in achieving optimum capture efficiency [42].

**Figure 3:**
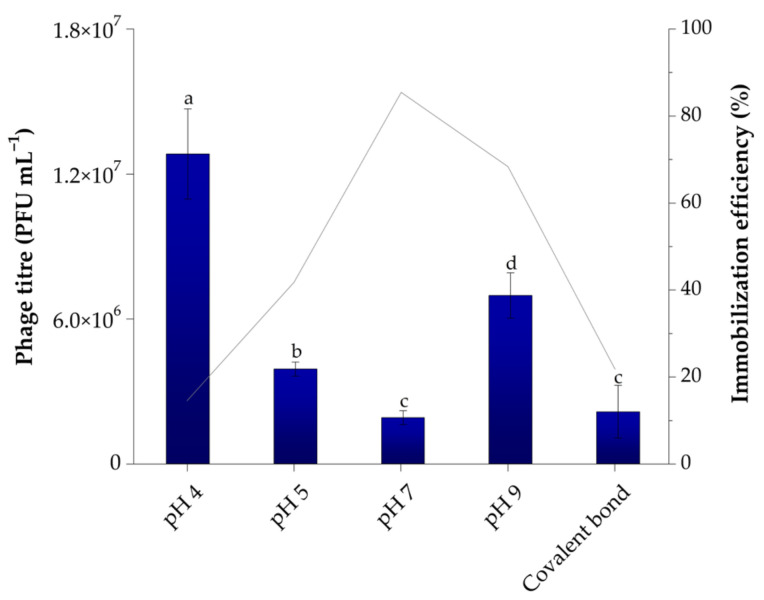
Phage activity (columns) and immobilization efficiency (solid line) of P100–MBs (MicroMod^®^, 32 µg) modified with a 10^9^ PFU mL^−1^ phage solution at the indicated pH values. Error bars denote the standard deviation from the mean of three independent experiments. Different lowercase letters represent significantly different mean values (*p* < 0.05).

Accordingly, besides the estimation of immobilization efficiency (%, by Equation (1)), the infectivity retention of P100–MBs was also considered in the optimization of the P100 immobilization protocol. Herein, the influence of the immobilization method (physical adsorption/electrostatic or covalent) and incubation solution pH effect on immobilized phage orientation was studied (Figure 3).

The overall results evidenced that physical immobilization has the potential to achieve a greater number of active phages (properly oriented) on PEG–MBs, rather than the non-oriented covalent bound protocol. The best P100 immobilization efficiency was achieved at pH 7 (77%) and the poorest results were obtained at pH 4 (38%), closer to the covalent protocol results (42%). In contrast, the best lytic activity level was observed at pH 4 suggesting a preferential charge-related “tail-upward” orientation of immobilized P100, which improved the probability of bacteria recognition, despite the lower phage concentration on its surface.

The DLS studies and P100 electric dipole moment conjugated with the MBs zeta-potentials (+ 6.42 and + 1.83 mV, at pH 4 and 5; data presented in Figure S1a) may support the obtained results. Nonetheless, the interaction of bacteriophages at solid–water interfaces is very complex and cannot be explained solely based on sorbent surface and phage isoelectric point, since hydrophobic effects and other minor interactions (e.g., hydrogen bonding, steric hindrance) can also favour P100 adhesion, albeit more weakly and reversibly than under electrostatic forces conditions [37,43]. A favourable contribution of this phenomenon was empirically observed in experiments performed at pH 7 and 9, which achieved high efficiency of phage immobilization despite the predicted low electrostatic forces.

Concerning the maintenance of P100 stability (aggregates formation at pH 4), and likely irreversible electrostatic physisorption, a pH 5 immobilization solution was selected for the optimized P100 immobilization protocol used in subsequent capture experiments [44]. Hereupon, stability studies of the optimized P100–MBs were also conducted, being disclosed remarkable stability to changes in ionic strength and pH immediately after physisorption (see immobilization protocol) and beyond (long-term storage), maintaining 90% of its initial lytic activity after 8 weeks.

### 3.2. Influence of Non-Specific Adsorption: Surface Blocking Step Optimization

The P100–MBs surface blocking step was optimized according to critical variables such as concentration and incubation time of the blocking solution. This study evaluated and compared two different standard blocking agents (BSA and casein) with distinct sizes and adsorption strengths on hydrophilic surfaces, to maximize the blocking of the unmodified sites of the PEG–MBs while ensuring a reduced steric hindrance of attached P100 [33,34].

**Figure 4:**
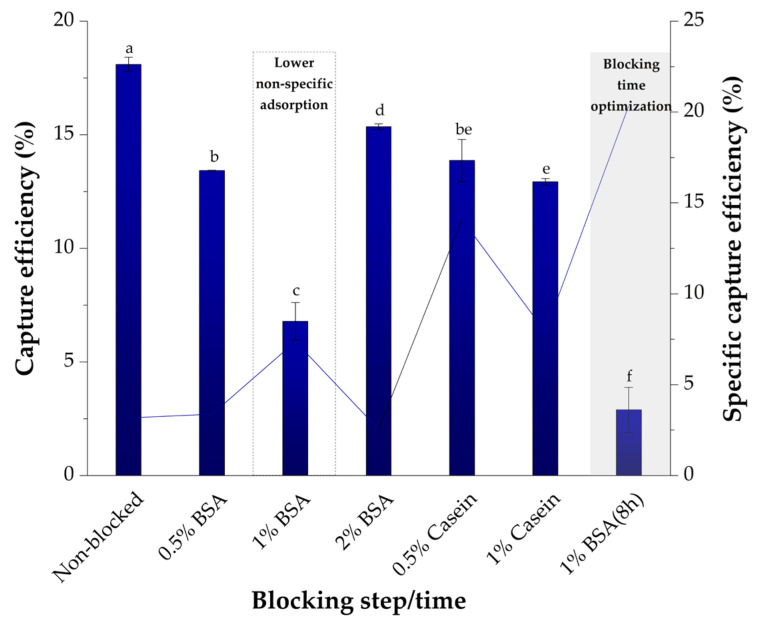
Effect of BSA and casein concentrations on the capture efficiency (columns) of blank PEG–MBs and the specific capture efficiency (solid line) of P100–MBs for *L. monocytogenes* (10^3^ CFU mL^−1^) separation, for a blocking time of 1 h (1% BSA, 1 h and 8 h). Error bars denote the standard deviation from the mean of four independent experiments. Different lowercase letters indicate statistical significance (*p* < 0.05).

Figure 4 illustrates the effects of BSA and casein concentration (%, *w*/*v*) on P100–MBs specific capture and non-specific adsorption, for a blocking time of 1 h and 8 h (grey shadow). The 1% BSA solution presented a positive effect in reducing non-specific adsorption (lower capture efficiency in blank assays with PEG–MBs) in 1 h step assays, besides exhibiting a relatively low specific capture compared to casein blocking protocols, even after increasing the BSA concentration to 2%. Contrastingly, casein was demonstrated to have a scarcer influence in reducing non-specific adsorption, compromising the selectivity of the developing separation method intended to be applied to complex food matrices. Henceforth, a 1% BSA concentration was selected for the blocking time optimization. The increasing blocking time (from 1 to 8 h), also had a positive effect on P100–MBs specific capture, presenting non-specific adsorption six-fold lower than unblocked blank PEG–MBs.

### 3.3. Phagomagnetic Separation Protocol Optimization

The optimization of phagomagnetic separation protocol experimental conditions (P100–MBs mass, pH, and temperature) was performed by resorting to the molybdophosphate culture-independent procedure. Briefly, this method is based on the reaction between phosphate moieties of bacterial DNA backbone with sodium molybdate to form an insoluble redox molybdophosphate precipitate, which may be electrochemically quantified and indirectly correlated to the bacterial load of the initial inoculum to calculate the specific capture efficiency (%) [27,45]. To test the practicability and viability of this approach, live and thermally lysed *L. monocytogenes* cells (10^3^ CFU mL^−1^) were electrochemically quantified using a disposable screen-printed carbon electrode (SPCE). The thermal-lysed cells solution showed two redox peaks at 0.19 V and 0.30 V, characteristics of the different valence states of molybdate present in the precipitate formed on SPCE [45,46,47]. Contrastingly, only residual redox peaks were displayed in the living cells solution (Figure S2 in Supplementary Materials), demonstrating the feasibility of the method to be used in the quantification of bacterial DNA released from the cells into the supernatant immediately after the phagomagnetic capture.

**Figure 5:**
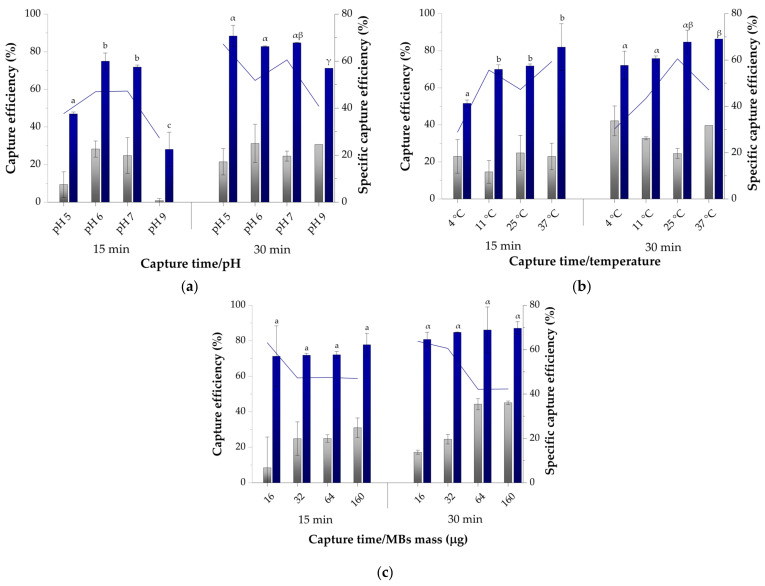
Phagomagnetic separation protocol optimization resorting to molybdophosphate method. The capture efficiency (%) (columns) and specific capture efficiency (%) (solid line) of P100–MBs (■) and blank–MBs (■) for the separation of *L. monocytogenes* (10^3^ CFU mL^−1^). Each variable was optimized following fixed assay conditions: (**a**) 32 µg MBs, 25 °C; (**b**) 32 µg MBs, pH 7; (**c**) 25 °C, pH 7. Each error bar was estimated as the standard deviation of three independent experiments. Different lowercase letters indicate statistical significance (*p* < 0.05).

The results of the phagomagnetic capture optimization are summarized in Figure 5 and unveiled a general increase in specific capture rates with longer capture times when comparing the 15 to 30 min protocols. Extended times were not tested to avoid interference from the lytic effect of P100 on the study.

Regarding the pH and MBs mass variables, alkaline incubation solutions (pH 9) and a high concentration of P100–MBs were demonstrated to impair the capture efficiency at low bacterial load. Thus, a mass between 16 and 32 µg is adequate for the contamination level evaluated (10^3^ CFU mL^−1^). Moreover, higher temperatures (25 and 37 °C) appear to promote a more effective capture. Therefore, an incubation solution of pH 7, a temperature of 25 °C, and a magnetic probes mass of 32 µg were selected as reference conditions, presenting a capture efficiency and specific capture of 80% and 60.6%, respectively (30 min of capture time). Zhou et al. [26] also evaluated the performance of magnetic particles biofunctionalized with the phage P100 (physically immobilized) and documented a significantly lower capture efficiency (40–50%) compared to the value obtained in the current work.

### 3.4. LAMP Assay Targeting prfA

We then sought to develop a novel LAMP system to be coupled with the previously optimized phagomagnetic separation platform towards the specific detection of viable *L. monocytogenes*. Following LAMP optimization, the performance of the method was validated through the specificity evaluation (inclusivity and exclusivity) and analytical sensitivity determination.

#### 3.4.1. Primers Efficiency Evaluation

An experiment was conducted to ascertain the most efficient LAMP primers set amongst the three sets of four oligonucleotides designed. Sets 2 and 3 were not capable of consistently hybridizing to the two on-target sequences assessed, irrespective of the temperature, and hence were excluded. Contrastingly, primer set 1 demonstrated a systematic specific recognition and hybridization to the cognate DNA templates of the *L. monocytogenes* strains (representatives of serotypes 1/2a, and 4b) evaluated, presenting the best compromise between non-specific background and amplification efficiency. In fact, this remarkable performance was inferred through the electrophoretic profile of the corresponding LAMP amplicons being observed, the characteristic ladder-like pattern (Figure S3, Supplementary Materials), hence validating the newly designed primer set selection to be utilized in the following experiments. Moreover, since no spurious amplicon formation was visualized, the negative control proved the non-occurrence of oligo primers dimerization or heterodimerization, corroborating the in silico prediction.

#### 3.4.2. Assessment of LAMP Specificity—Inclusivity

The optimized LAMP procedure proved the capability to robustly identify a 61-cohort of *L. monocytogenes* strains belonging to each of the three most common invasive listeriosis-associated serotypes (1/2a, 1/2b, 4b), along with serotype 1/2c (Table 1). The bacterial cohort comprised strains belonging to the genetic lineage I (harbouring serotypes 1/2b and 4b), and lineage II (comprising serotypes 1/2a and 1/2c).

**Figure 6:**
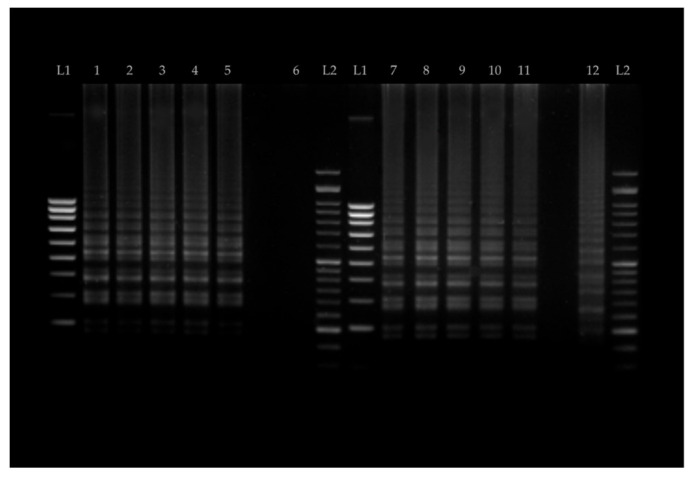
Inclusivity of the LAMP assay evaluated through agarose gel electrophoresis. The experiments were conducted at 62 °C (lanes 1 to 6) and 63 °C (lanes 7 to 12). Lanes 1 and 7, *Lm* 1/2a; lanes 2 and 8, *Lm* 4b; lanes 3 and 9, *Lm* 1/2b; lanes 4 and 10, *Lm* 1/2c; lanes 5 and 11, *Lm* reference strain (EGDe); lanes 6 and 12, non-target DNA control. Lanes L1 and L2, molecular weight marker (NZYDNA Ladder V and VI).

The electrophoretic analysis disclosed the remarkable inclusivity (100%) of the developed assay since a conspicuous amplicons profile (owing to the formation of the stem-loop DNA structures) was visualized (Figure 6), highlighting the positive on-target DNA amplification of the four serotypes considered. The specific detection of different strains belonging to the same serotype was highly consistent and supported that this conserved gene is an appropriate target for the broad-spectrum identification of *L. monocytogenes*. Cho et al. [48] also investigated the feasibility of the isothermal method targeting *prfA* for *L. monocytogenes* detection and the results, in agreement with the documented herein, underlined the high specificity (100% of inclusivity) of the assay, since the amplicons of the 23 *L. monocytogenes* strains assessed were systematically generated.

LAMP results were in accordance with the positive signal obtained with the conventional PCR. D’Agostino et al. [30], utilizing the same PCR oligonucleotides, evaluated the assay performance against a panel of 38 *L. monocytogenes* and documented the notable efficiency (100% inclusive) of the method. Consistent with our findings, Cooray et al. [49] also reported the suitability of utilizing *prfA* as a highly species-specific gene.

#### 3.4.3. Evaluation of LAMP Specificity—Exclusivity

The potential cross-reactivity of the proposed LAMP assay was further examined. The electrophoretic pattern obtained (Figure S4, Supplementary Materials) demonstrated that the LAMP reaction system is highly species-specific, and cross-hybridization was not observed for the other closely related *Listeria* species, namely *Listeria sensu stricto* species, *Listeria ivanovii* NCTC 11846 and *Listeria innocua* 2030c, and *Listeria sensu lato* species, *Listeria aquatica* [50]. Noteworthy, the occurrence of the *prfA* gene is not constrained to the virulent *L. monocytogenes* strains, since *L. ivanovii* NCTC 11846 (the animal pathogen) harbours an orthologous gene (albeit with a low nucleotide sequence similarity to the query DNA conserved region), whilst the non-pathogenic environmental saprophyte *L. aquatica* is devoid of the whole *prfA* gene cluster [51]. LAMP results corroborated the previous in silico prediction of the absence of hybridization of the designed oligonucleotides with the heterologous *L. ivanovii* NCTC 11846 DNA sequence.

Moreover, no apparent cross-amplifications were noticed for all the 39 non-*Listeria* strains (20 Gram-positive and 19 Gram-negative bacteria) tested. These observations evince the non-formation of the characteristic “dumbbell” structures, indicative of no nonspecific complementarity of the oligonucleotides with the reference non-target DNA sequences. Concerning the experiments performed at a higher temperature (63 °C), the non-template DNA displayed a faint electrophoretic profile (Figure 6, lane 12) which was evincive of spurious hybridization. Therefore, 62 °C was deemed to be the optimum temperature since an improved stringency was accomplished. This LAMP method was found to be 100% exclusive towards 42 non-target Gram-positive and Gram-negative bacterial strains.

Pertaining to the PCR specificity (Table 1), the results were in close agreement with the proposed LAMP assay, corroborating the formerly documented by Simon et al. [29] and D’Agostino et al. [30]. According to the latter, amongst the 52 non-*L. monocytogenes* strains evaluated, the *prfA*-based PCR method proved 100% exclusivity.

In opposition to the PCR and RT–PCR-based approaches targeting *prfA* [52,53,54,55], hitherto *prfA*-based LAMP assays have been scarcely exploited. Cho et al. [48] also assessed LAMP specificity towards 16 non-*L. monocytogenes* strains and the method demonstrated 100% of exclusivity, which is in close agreement with the reported herein. Considering our findings and those formerly documented in the previous study, one may conclude that *prfA* is, as aforementioned, an appropriate target gene towards the specific LAMP detection of *L. monocytogenes*.

#### 3.4.4. Evaluation of LAMP Analytical Performance (LOD_95_)

The analytical sensitivity (limit of detection) of the newly developed LAMP assay was also investigated. Probit analysis was conducted to estimate the LOD of the designed LAMP (Figure S5 in Supplementary Materials) with 95% of confidence (LOD_95_), being obtained a value of 1.98 fg μL^−1^ (0.95 level of confidence interval 1.1 to 15 fg μL^−1^), theoretically equivalent to 0.5 CFU mL^−1^. Hence, the current method was demonstrated to be highly sensitive, since it proved efficient in consistently detecting as few as 0.063 copies of the genome per reaction (1.98 fg μL^−1^ of *L. monocytogenes* genomic DNA).

For comparative purposes, the sensitivity performance of conventional PCR was also assessed using the same tenfold standard dilutions of template DNA. In opposition to the LAMP assay, an order-of-magnitude higher LOD_95_ was obtained, indicating that the latter was 20-fold more sensitive. Moreover, LAMP DNA amplification was accomplished 42 min swifter than the standard PCR.

Table S2 (Supplementary Materials) outlines important features of LAMP protocols compiled through a systematic literature review pertaining to the highly specific *L. monocytogenes* detection. The probit estimated LOD_95_ (0.5 CFU mL^−1^) was within the same order of magnitude as those values documented by Wachiralurpan et al. [56] and Lee et al. [15], which developed LAMP protocols with the capability of detecting as low as 0.3–3 and 1 CFU mL^−1^, respectively. The comparison with other detection thresholds gathered from the available literature (Table S2) highlighted the superior analytical performance of the current method, which displayed a LOD_95_ value 6- to 20,000-fold lower [14,15,17,57,58,59,60].

Concerning the paramount importance of the oligo primers length, Wachiralurpan et al. [17] hypothesized that the 1000-fold difference in the LOD value of LAMP assays targeting *plcB* (2.8 CFU mL^−1^) and *hly* (2.8 × 10^3^ CFU mL^−1^) might be attributed to the longer sequence of the latter, which possessed a lower annealing efficiency owing to the putative formation of secondary structures.

In another study (in which *actA* was targeted), in an attempt to accomplish a higher detection performance [14], a pre-concentration approach was proposed, attaining a 10-fold lower cut-off value when aptamer-based magnetic capture was associated with LAMP. Moreover, in most works, the LAMP technique has proven to surpass PCR analytical sensitivity (by at least ten-fold), corroborating the herein-obtained results.

The detection limit and selectivity of these molecular-based approaches are considered pivotal parameters to evaluate the accuracy of the method [30,61]. These features highlighted the superior performance of the herein proposed LAMP technique, substantiating the suitability of this method as an affordable routine screening procedure for the presumptive presence of *L. monocytogenes*. With the development and optimization of the current LAMP procedure, the groundwork was laid for the validation of its applicability in food matrices.

### 3.5. Applicability of the Combined Detection System in Pasteurized Milk

#### 3.5.1. Phagomagnetic Particles Performance in Pasteurized Milk

The inherent complexity of milk composition poses a challenge in the development of effective foodborne pathogens detection protocols, since some components (protein and lipid content) may constitute critical interferents in magnetic separation procedures.

**Figure 7:**
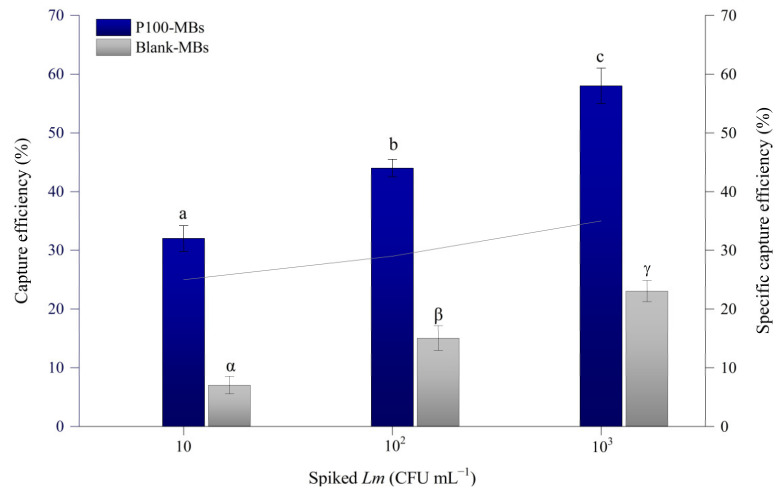
Phagomagnetic separation of *L. monocytogenes* from spiked (10–10^3^ CFU mL^−1^) pasteurized milk utilizing P100–MBs (32 µg, 25 °C), following 30 min of incubation. Capture efficiency (%) (columns) and specific capture efficiency (%) (solid line) were determined. Each error bar was estimated as the standard deviation of three independent experiments. Different lowercase letters indicate statistical significance (*p* < 0.05).

Hence, the previously optimized phagomagnetic separation protocol was exploited to evaluate the *L. monocytogenes* capture performance in pasteurized whole milk. The capture efficiency value (58%, Figure 7) of P100–MBs in milk samples spiked with 10^3^ CFU mL^−1^ was lower than the value obtained at 30 min in Tris buffer pH 7.2 (85%).

Results disclosed a 2.5-fold enhancement in the *L. monocytogenes* capture for P100–MBs in comparison to the corresponding phage-devoid MBs counterpart, demonstrating the ability of the immobilized phage to capture bacterial cells. Moreover, the P100–MBs were found to be highly sensitive, presenting a separation limit below 10 CFU mL^−1^, with a significant increase (*p* < 0.05) in the capture efficiency, concomitantly with specific capture efficiency, up to 10^3^ CFU mL^−1^ (Figure 7).

According to the aforementioned results (Section 3.3), the interaction of *L. monocytogenes* and P100–MBs was not pH-dependent. Therefore, the lower performance of P100–MBs in milk may be attributed to the protein and lipid contents of the matrix. Those may constitute pivotal interferents in the phagomagnetic particle diffusion, hence influencing the P100–MBs adsorption to the target bacterium. The milk proteins (casein (insoluble) and whey proteins (soluble)) may hinder the contact between immobilized phages and *L. monocytogenes* [62]. Concerning the milk lipid content, the occurrence of electrostatic and hydrophobic interactions between PEG-immobilized virion particles and lipid molecules may also hamper the bacterium attachment [63].

Zhou et al. [26] also exploited phage P100 as a biorecognition element to propose a phagomagnetic protocol towards *L. monocytogenes* isolation in whole milk. The authors documented a significantly lower capture efficiency (46%) in comparison to the value obtained herein (58%).

In a distinct approach, Shan et al. [19] proposed an immunomagnetic method to isolate the same bacteria in whole milk, being reported a higher separation performance (85%). Notwithstanding, in the current work a lower separation limit was attained (10 CFU mL^−1^) in comparison to the determined therein (10^3^ CFU mL^−1^). Accordingly, Yang et al. [11] also employing immunomagnetic nanoparticles in semi-skimmed milk, documented a low sensitivity of the separation method (10^2^ CFU mL^−1^) with a low capture efficiency (4.6% for a bacterial load of 10^2^ CFU mL^−1^).

A novel pre-concentration platform relying on ampicillin-biofunctionalized magnetic nanoparticles was recently described by Bai et al. [64] and a higher limit of detection in spiked milk was reported (10^2^ CFU mL^−1^), even when in combination with qPCR. Noteworthy, owing to the broad-spectrum activity of the bioreceptor, a low specificity was observed.

The phagomagnetic method proposed herein demonstrated to be a promising bio-approach to selectively capture and pre-concentrate *L. monocytogenes* in pasteurized whole milk. In particular, the results convey the utility of this platform, which holds a remarkable potential to isolate VBNC *L. monocytogenes* cells from a complex food matrix for accurate downstream detection.

#### 3.5.2. Phagomagnetic-Assisted LAMP Assay

The efficiency of the optimized LAMP assay was assessed concerning the detection of the *Lm*-P100–MBs complex, previously isolated from spiked pasteurized milk. Despite the notable specificity of the LAMP technique, one of the claimed drawbacks is the inability to distinguish viable virulent cells from dead harmless analogues. Hence, owing to this lack of discriminatory potential of the DNA amplification technique, biased *L. monocytogenes* detection results might occur, leading to false positive detection. The coupling of LAMP with a prior phagomagnetic capture addressed this issue.

Moreover, we sought to exploit an alternative method (phage P100-mediated lysis) to the classic DNA extraction procedures. The rationale underlying the dual-purpose (capture and lysis) phage-based approach relied on the notable potential of this virus as a biorecognition element to specifically adsorb to viable *L. monocytogenes* cells, along with its intrinsic strict lytic trait, triggering the ensuing bacterial chromosomal DNA leakage.

**Figure 8:**
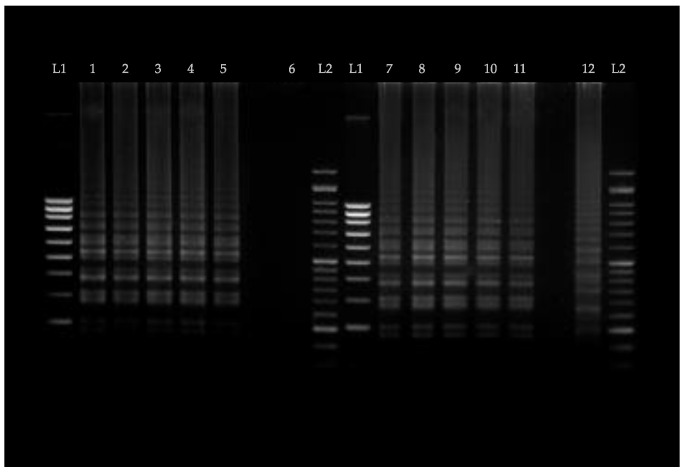
Evaluation of the LAMP-amplified DNA obtained following P100–MBs mediated lysis of *L. monocytogenes* isolated from pasteurized milk and DNA extracted from pure bacterial cultures. Lanes: 1,6—P100–MBs mediated lysis of *L. monocytogenes* -10^3^ CFU mL^−1^; 2,7—10^2^ CFU mL^−1^; 3,8—10 CFU mL^−1^; 4,9—5 CFU mL^−1^; 5,10—1 CFU mL^−1^; NTC—non-template control (10^3^ CFU mL^−1^); NC—negative control. Lanes L1 and L2, molecular weight marker (NZYDNA Ladder VI and V).

The P100–MBs mediated lysis of *L. monocytogenes* isolated from pasteurized milk or culture medium was electrophoretically assessed. The analysis disclosed the effective P100-induced lysis of magnetically captured *L. monocytogenes* since leaked genomic DNA was observed for the distinct bacterial loads analysed (5 to 10^2^ CFU mL^−1^) (Figure 8 and Figure S6, Supplementary Materials). Moreover, the efficiency of detecting the host DNA in phage lysates of cells captured from milk or isolated from cells in a culture medium was comparable (Figure S6). Additionally, the absence of DNA in the *Lm*-blank–MBs sample (phage-negative control) supported that the nucleic acid was released owing to the specific phage infection, highlighting the outstanding lytic performance of phage P100. Therefore, the proposed method proved the suitability to preclude the use of a nucleic acid isolation kit, which is a prominent advantage. Noteworthy, as aforementioned, this phage-based approach warrants the detection of viable *L. monocytogenes* and hence provides high confidence for the confirmation of the contamination.

The results obtained herein were in close accordance with former studies [12,24,64]. Tlili et al. [24] were the first to exploit a phage-mediated lysis protocol to extract genomic DNA from bacterial host cells. The authors reported that the phage T4 covalently immobilized onto the surface of a gold electrode elicited the irreversible intracellular DNA delivery of the T4-captured *E. coli* into the lysate milieu. The target gene *tuf* was subsequently LAMP-amplified and detected via linear sweep voltammetry. Wang et al. [12] proposed an experimental merged scheme analogous to the presented herein, in which a coliphage, covalently conjugated with magnetic beads, was utilized as a bioreceptor and lysing agent for viable *E. coli* O157:H7. The extracellularly leaked bacterial DNA was amplified by qPCR to quantify and identify the target bacterium in water samples. Swift et al. [65] developed a mycobacterium phage D29-triggered lysis (Actiphage^®^) procedure to efficiently extract genomic DNA from viable, low-cell numbers, mycobacteria. The released bacterial DNA was subsequently utilized as the template for PCR amplification, providing a sensitive detection tool for viable and pathogenic mycobacteria collected from blood specimens.

The method proposed herein circumvents the use of laborious commercial DNA extraction kits, which is of utmost importance to accomplish the straightforwardness and cost-efficiency required for an on-field application. Furthermore, the inclusion of the magnetic capture step proved to be appropriate to efficiently cope with the inhibitors/interferents estimated to be present in the pasteurized milk sample.

#### 3.5.3. Detection Limit of the Phagomagnetic-Assisted LAMP Assay in Milk

The performance of the novel LAMP assay in pasteurized milk was evaluated by electrophoretic analysis, and the analytical sensitivity (LOD_95_) was determined by probit regression (Figure S7, in Supplementary Materials). The current method was determined to be highly sensitive since it proved efficient in consistently detecting as few as 5 CFU mL^−1^ (LOD_95_ of 4.1 CFU mL^−1^). In comparison to the LAMP amplification performed with high-purity genomic DNA extracted (commercial kit) from *L. monocytogenes* pure cultures, devoid of prior magnetic isolation and subsequent phage-mediated lysis, a 10-fold lower sensitivity was obtained. One may theorize that this discrepancy may be attributed to the magnetic platform’s inefficiency in capturing the totality of the bacterial load.

**Figure 9:**
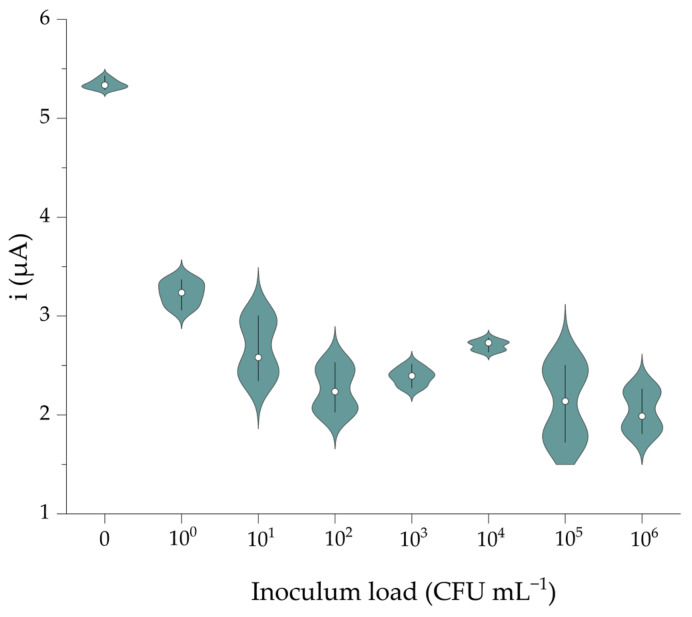
Endpoint electrochemical detection (square wave voltammetry) of LAMP amplicons obtained following P100–MBs mediated isolation and lysis of *L. monocytogenes* from spiked pasteurized milk.

Beyond the well-documented high specificity of the developed isothermal amplification technique, the versatility of the reaction readout, which may be performed (alternatively to the electrophoretic analysis) via an endpoint electrochemical technique resorting to MBlue intercalation, was explored. The voltametric analysis (Figure 9) disclosed that the technique presented the potential to detect *L. monocytogenes* with high analytical sensitivity (1 CFU mL^−1^) in 20 min, which proved a superior detection performance compared to the electrophoretic analysis (5 CFU mL^−1^).

Accordingly, Lau et al. [66] and Azek et al. [67] also documented an improved analytical sensitivity of electrochemical detection techniques, over conventional gel electrophoresis. The improved efficiency (combined with the swiftness and convenience) of electrochemical readouts may contribute to the suitable implementation of this rapid detection method in resource-scarce industries. This on-time surveillance system would be highly valuable, with a remarkable potential for practical application in the dairy industry.

Amongst the detection thresholds gathered from the available literature on LAMP-based detection of *L. monocytogenes* in milk (Table S3 in Supplementary Materials), solely Roumani et al. [20] accomplished a significantly lower value (0.11 CFU g^−1^) than the documented herein. One may surmise that the superior sensitivity could be attributable to the 24h-selective enrichment of the spiked milk (before the analytical phase), hence eliciting the enhancement of the initial bacterial load of the matrix which, therefore, may warrant the biased (improved) LAMP detection of such a low number of *L. monocytogenes* cells. The value obtained in this work is in close accordance with the LOD_95_ of formerly developed LAMP procedures [48,68,69] with values of the same order of magnitude (1–3.2 CFU mL^−1^). Contrastingly, evaluation of the analytical performance of the current assay indicated a considerable superiority compared with LAMP methods proposed by Wang et al. [70], Teixeira et al. [71], and Wang et al. [72], displaying a limit of detection value 45-, 90- and 6000-fold lower, respectively. Accordingly, a 2000-fold sensitivity improvement was achieved in comparison to the commercially available LAMP kit for *L. monocytogenes* detection (Eiken), which is constrained by a limit of detection of 10^4^–10^5^ CFU mL^−1^.

## 4. Conclusions

In the current work, we demonstrated the feasibility of coupling a novel targeted LAMP assay (assisted by a P100–MB platform) with an endpoint electrochemical readout system towards a swift and accurate *L. monocytogenes* detection in food matrices. This system succeeded in the accomplishment of the requirements considered pivotal for the implementation of an on-site detection method, namely the needlessness of robust and sophisticated laboratory apparatus, the non-inclusion of a lengthy culture-based selective enrichment protocol, an expeditious procedure (2.5 h), and a notable sensitivity (1 CFU mL^−1^). Moreover, the proposed combined approach may provide a reliable molecular-based surveillance tool for food safety analytical services and public health authorities. The developed detection scheme could be of utmost importance for the demonstration/validation of compliance with food safety standards.

## Figures and Tables

**Table 1:** Bacterial strains used for LAMP evaluation and PCR validation.

	Bacterial Species and Serotype	Source	No. of Strains	LAMP Result	PCR Result
*Listeria* spp.	*L. monocytogenes*	ATCC BAA-679	1	+	+
	*L. monocytogenes* (1/2a)	CCESB	15	+	+
	*L. monocytogenes* (1/2b)	CCESB	15	+	+
	*L. monocytogenes* (1/2c)	CCESB	15	+	+
	*L. monocytogenes* (4b)	CCESB	15	+	+
	*L. innocua* 2030c	PHLS	1	-	-
	*L. ivanovii*	ATCC 19119	1	-	-
	*L. aquatica*	CCESB	1	-	-
*Enterococcus* spp.	E. faecalis	ATCC 29212	1	-	-
	*E. faecalis*	CCESB	3	-	-
	*E. faecium*	DSMZ 13590	1	-	-
	*E. faecium*	CCESB	2	-	-
*Staphylococcus* spp.	*S. aureus*	ATCC 29213	1	-	-
	*S. aureus*	ATCC 25923	1	-	-
	*S. aureus*	CCESB	6	-	-
*Lactococcus* spp.	*L. lactis*	DSMZ 4366	1	-	-
	*L. lactis*	CCESB	2	-	-
*Leuconostoc* spp.	*L. mesenteroides*	CCESB	2	-	-
*Pseudomonas* spp.	*P. aeruginosa*	ATCC 27853	1	-	-
*Escherichia* spp.	*E. coli*	ATCC 25922	1	-	-
*Salmonella enterica*	*S.* Infantis M2016	NFCSO	1	-	-
	*S.* Braenderup	ATCC BAA-664	1	-	-
	*S.* Weltevreden TA 428/97	EURL	1	-	-
	*S.* Senftenberg	ATCC 43845	1	-	-
	*S.* Typhimurium	ATCC 14028	1	-	-
	*S.* Derby	CCESB	1	-	-
	*S.* Enteritidis	ATCC 13076	1	-	-
	*S.* Wernigerode	CCESB	1	-	-
*Acinetobacter* spp.	*A. baumannii*	CCESB	2	-	-
*Campylobacter* spp.	*C. jejuni*	DSMZ 4688	1	-	-
	*C. coli*	DSMZ 4689	1	-	-
	*C. lari*	DSMZ 11375	1	-	-

ATCC—American Type Culture Collection; DSMZ—German Collection of Microorganisms and Cell Culture; EURL—European Union Reference Laboratory; CCESB—Culture collection of Escola Superior de Biotecnologia; NCTC—National Collection of Type Cultures; NFCSO—National Food Chain Safety Office strain collection; PHLS—Public Health Laboratory Service.

## Data Availability

Data is contained within the article or Supplementary Materials.

## Supplementary Materials

The following supporting information can be downloaded at: https://www.mdpi.com/article/10.3390/bios13040464/s1, Section S1: pH Buffer solutions and culture medium preparation; Figure S1: (a) Zeta potential of PEG-NH_2_-MPs (MicroMod®, ⌀ 2 µm) *versus* dispersion solution pH (0.01M citrate buffer for pH 4 and 5, and 0.01M PBS for pH 7, 9, and 11). (b) Intensity distribution *versus* hydrodynamic diameter for phage P100 at the indicated pH values.; Table S1: Sequences of the LAMP and PCR oligo primers; Figure S2: Square-wave voltammograms of living and thermally lysed *L. monocytogenes* cells (10^3^ CFU mL^−1^) using the molybdophosphate culture-independent protocol and a disposable SPCE; Figure S3: Evaluation of the oligo primers efficiency for LAMP assay targeting *prfA* through gel electrophoresis analysis of loop isothermal amplified products. L1 and L2, DNA ladder (NZYDNA Ladder V and VI); 1,4,7,10,13,16-*Lm* 1/2a; 2,5,8,11,14,17-*Lm* 4b; 3,6,9,12,15,18-Negative control (nuclease-free water); Figure S4: Exclusivity of the LAMP assay (62 °C) evaluated through agarose gel electrophoresis. Lanes 1 through 5, on-target *L. monocytogenes*; lane 6, *L. ivanovii* NCTC 11846; lane 7, *L. innocua* 2030c; lane 8, *L. aquatica*; lane 8, *E. faecalis* ATCC 29212; lane 9, *S. aureus* ATCC 29213; lane 10, *L. lactis* DSMZ 4366; lane 11, *L. mesenteroides*; lane 12, *E. coli* ATCC 25922; lane 13, *S. enterica* serovar Typhimurium ATCC 14028; lane 14, *P. aeruginosa* ATCC 27853; lane 15, *C. jejuni* DSMZ 4688; NC, negative control. Lanes L1 and L2, molecular weight marker (NZYDNA Ladder V and VI); Figure S5: Sensitivity of LAMP procedure for detection of *L. monocytogenes* by probit regression; Table S2: Analysis of the analytical sensitivity (LOD) of LAMP assays for *L. monocytogenes* detection documented in the literature; Figure S6: Evaluation of P100-MBs mediated lysis of *L. monocytogenes* isolated from pasteurized milk and pure culture. Lanes: L1—DNA ladder; 1—*L. monocytogenes*-blank-MBs (negative control-P100 devoid, 10^3^ CFU mL^−1^); 2-7 – P100-MBs mediated lysis of *L. monocytogene*s: 10^2^ CFU mL^−1^ (2,5), 10 CFU mL^−1^ (3,6), 5 CFU mL^−1^ (4,7); Figure S7: Results of probit analysis in milk samples—LOD_95_; Figure S8: (A) Peak currents of MBlue at the indicated concentrations (5–25 mM) obtained in 0.01M Tris pH 7.2 with 0.02M KCl; (B) Graphical representation of the average values of peak current (*n* = 4) *versus* MBlue concentration; (C) Peak current variation (average values with standard deviation (*n* = 4)) for different concentrations of MBlue solution upon mixing 1:1 with LAMP amplified DNA (10^5^ CFU mL^−1^). Different lowercase letters indicate statistical significance (*p* < 0.05). The square wave scans were obtained with 0.025 V amplitude, 0.004 V step potential and 100 Hz frequency; Table S3: Analysis of the literature on LAMP detection of *L. monocytogenes* in milk. References [72,73,74,75,76,77,78,79,80,81,82,83] are cited in the Supplementary Materials.
