# Supplementary material for: Development of a Novel Phagomagnetic-Assisted Isothermal DNA Amplification System for Endpoint Electrochemical Detection of Listeria monocytogenes
Source: Biosensors (Basel). 2023 Apr 7;13(4):464. doi: 10.3390/bios13040464 (PMC10136355; doi:10.3390/bios13040464)
Supplement: Supplementary file 1 [file biosensors-13-00464-s001.zip › biosensors-2300624-SI.pdf]

Development of a novel phagomagnetic-assisted isothermal DNA amplification system for endpoint electrochemical detection of *Listeria monocytogenes*

S1- pH Buffer solutions and culture medium preparation

0.01M PBS (338 mg $Na_2HPO_4.2H_2O$, 360 mg NaH_2PO_4, 4 g NaCl in 500 mL H_2O), pH adjustment to pH 7, 7.4, 9 and 11 with 0.1/1M NaOH. 0.01M PBST (0.01M PBS, pH 7.4 coupled with 0.01% Tween 20). 1M Tris pH 7.5 (260.57 g in 500 mL H_2O) pH adjustment with 1M HCl. SM buffer (2 g $MgSO_4.7H_2O$, 5.8 g NaCl, 1 mL gelatine from porcine skin (10% w/v) in 50 mL 1M Tris pH 7.5). 0.1M Tris pH 7.2 (26.06 g in 500 mL H_2O) pH adjustment with 0.1M HCl. 0.01M citrate buffer (1.05 g citric acid and 3.742 g NaCl in 450 mL H_2O, pH adjustment with 0.1M HCl or 1M NaOH). For the electrochemical detection of methylene blue, 0.01M Tris pH 7.2 with 0.02 M KCl was used. All solutions used throughout the work were previously filtered with a 0.2 μm sterile filter.

LC soft agar culture medium (10 g L^{-1} tryptone, 5 g L^{-1} yeast extract, 10 g L^{-1} glucose, 7.5 g L^{-1} NaCl, 10mM $MgSO_4$, 10mM $CaCl_2$, 0.4% (w/v) agar).

S2- Magnetic particles zeta potential and phage P100 dynamic light scattering (DLS) *versus* pH

The zeta potential and DLS experiments were performed at room temperature using a Malvern Zetasizer® (Nano ZS series). The isoelectric point of bare PEG-NH_2-MPs suspensions (20 μg mL^{-1}) was determined by analyzing the zeta potential against varying pH values (4 to 11) and the results are summarized in Figure S2a. The aggregation and hydrodynamic behaviour of P100 ($2x10^9$ plaque forming units (PFU) mL^{-1}) at varying pH values (2.5 to 9) was also studied and the results are summarized in Figure S2b.

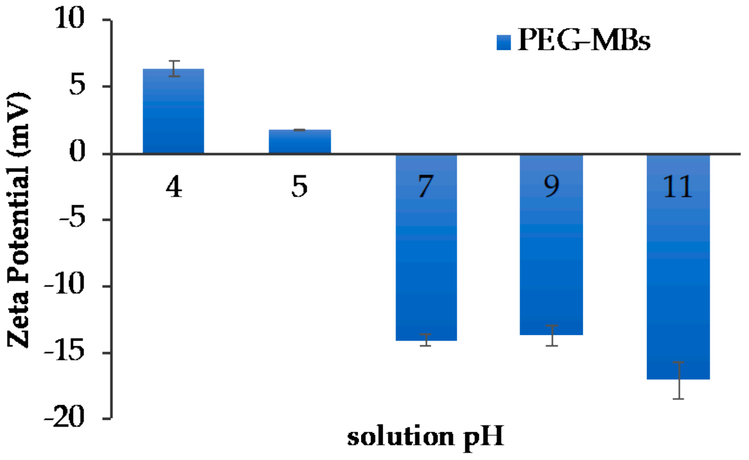

Figure S2a. Zeta potential of PEG-NH_2-MPs (MicroMod®, ⌀ 2 μm) *versus* dispersion solution pH (0.01M citrate buffer for pH 4 and 5, and 0.01M PBS for pH 7, 9, and 11).

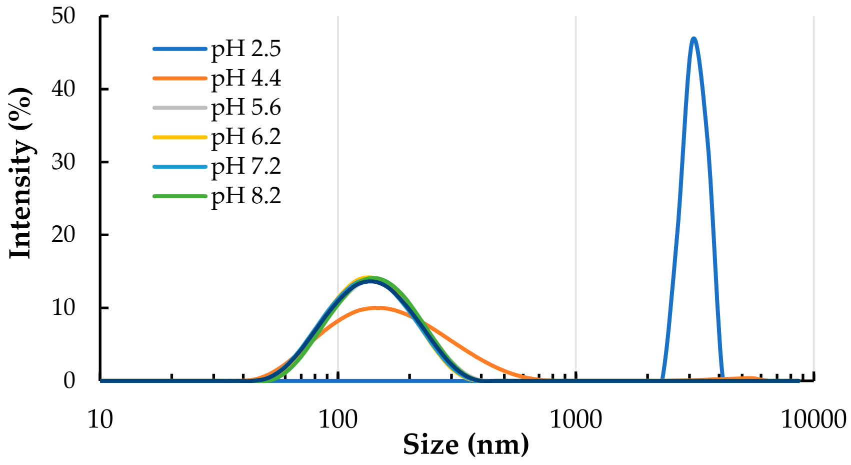

Figure S2b. Intensity distribution *versus* hydrodynamic diameter for phage P100 at the indicated pH values.

S3- Sequences of the LAMP and PCR oligo primers

Table S3. Sequences of the LAMP and PCR oligo primers used in the current work.

Primers		Sequence (5'-3')	Reference
PCR Primers			
LIP1		GATACAGAAACATCGGTTGGC	Simon et al., 1996 [29]
LIP2		GTGTAACTTGATGCCATCAGG	
LAMP Primers			
PrfA-F3		ACTACTGAGCAAAAATCTTACG	This work
PrfA-B3		ATCCTAACTCCTGCATTGT	
PrfA-FIP (F1c-F2)	F1c	GCTTCCCGTTAATCGAAAAATCATT-	
	F2	CACTTTTTCTATGTTTTCCAAACC	
PrfA-BIP (B1c-B2)	B1c	TGGCTCTATTTGCGGTCAAC -	
	B2	CAGTGTAATCTTGATGCCATC	

S4- Square-wave voltammograms of living and thermally lysed *L. monocytogenes* cells

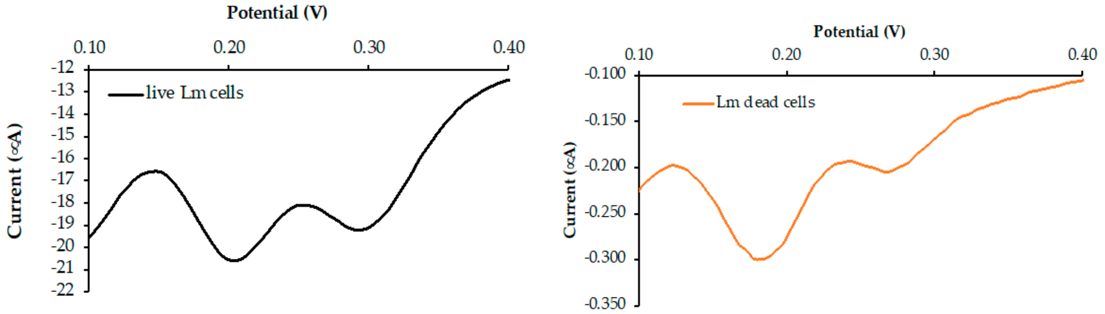

Figure S4. Square-wave voltammograms of living and thermally lysed *L. monocytogenes* cells (10^3 CFU mL^{-1}) using the molybdophosphate culture-independent protocol and a disposable SPCE.

S5- Evaluation of the oligo primers efficiency for LAMP assay targeting *prfA*

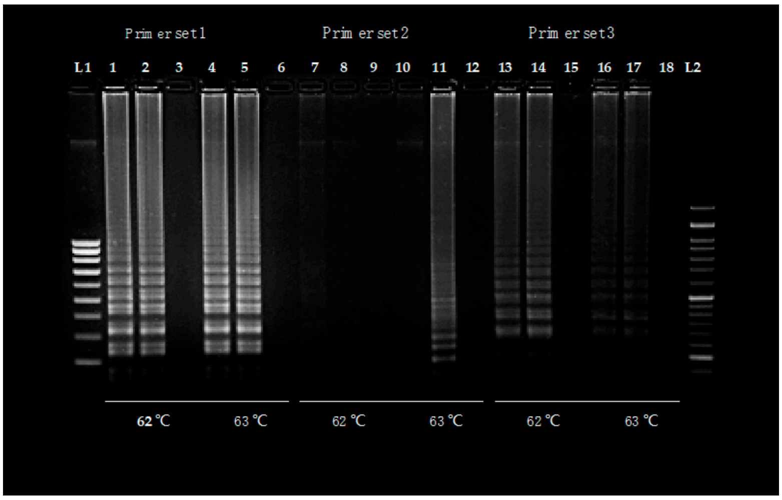

Figure S5. Evaluation of the oligo primers efficiency for LAMP assay targeting *prfA* through gel electrophoresis analysis of loop isothermal amplified products. L1 and L2, DNA ladder (NZYDNA Ladder V and VI); 1,4,7,10,13,16-*Lm* 1/2a; 2,5,8,11,14,17-*Lm* 4b; 3,6,9,12,15,18-Negative control (nuclease-free water).

S6- Exclusivity of the LAMP assay targeting *prfA*

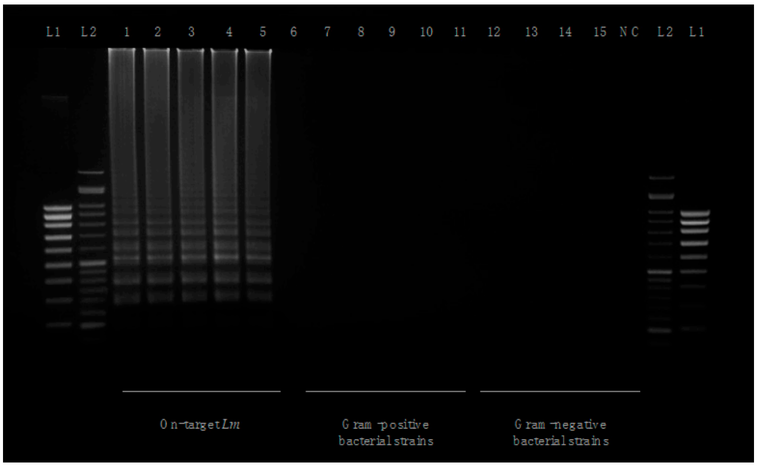

Figure S6. Exclusivity of the LAMP assay (62 °C) evaluated through agarose gel electrophoresis. Lanes 1 through 5, on-target *L. monocytogenes*; lane 6, *L. ivanovii* NCTC 11846; lane 7, *L. innocua* 2030c; lane 8, *L. aquatica*; lane 8, *E. faecalis* ATCC 29212; lane 9, *S. aureus* ATCC 29213; lane 10, *L. lactis* DSMZ 4366; lane 11, *L. mesenteroides*; lane 12, *E. coli* ATCC 25922; lane 13, *S. enterica* serovar Typhimurium ATCC 14028; lane 14, *P. aeruginosa* ATCC 27853; lane 15, *C. jejuni* DSMZ 4688; NC, negative control. Lanes L1 and L2, molecular weight marker (NZYDNA Ladder V and VI).

S7- Results of probit analysis - LOD$_{95}$

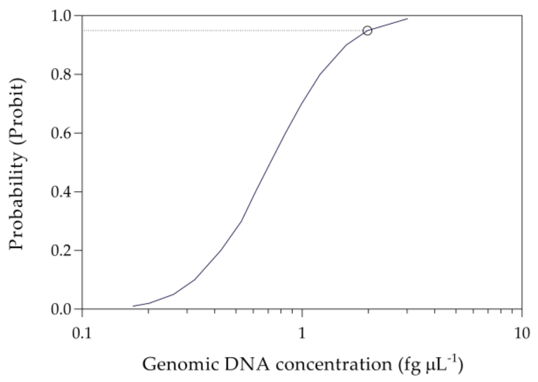

Probability	95% Confidence Limits for fg µL^{-1}		
	Estimate	Lower Bound	Upper Bound
0.01	0.17	0.04	0.26
0.05	0.26	0.11	0.36
0.10	0.33	0.18	0.44
0.20	0.43	0.29	0.60
0.30	0.52	0.38	0.82
0.40	0.61	0.46	1.13
0.50	0.72	0.53	1.56
0.60	0.84	0.60	2.18
0.70	0.99	0.68	3.15
0.80	1.21	0.78	4.87
0.90	1.59	0.94	9.01
0.95	1.98	1.10	15.02
0.99	3.03	1.45	39.39

Sensitivity of LAMP procedure for detection of *L. monocytogenes* by probit regression.

Table S8. Analysis of the analytical sensitivity (LOD) of LAMP assays for *L. monocytogenes* detection documented in the literature.

LAMP format	Target gene	Detection approach	Test matrix	Detection time	LOD	Reference
LAMP	*hlyA*	Phosphate precipitation and calcein	Nuclease free water	50 min (LAMP)	1 x 10^3 CFU mL^{-1}	[72]
PMA-LAMP	*hlyA*	Fluorescence	BHI inoculated	2.5 h min (45 min LAMP)	3.1 x 10^2 CFU mL^{-1}	[73]
LAMP	*hlyA*	Colorimetric and Fluorescence	Tris-HCl pH 8, 1mM EDTA	50 min (LAMP)	1 x 10^4 CFU mL^{-1}	[59]
LAMP	*hly*	Electrophoresis	Luria Broth (LB) inoculated	4.5 h (30 min LAMP)	100 CFU	[74]
On-chip LAMP		Fluorescence				
RT-LAMP	*hly*	Turbidity	BHI inoculated	60 min LAMP	2.82 x 10^3 CFU mL^{-1}	[56]
LAMP-LFD	*plcB*	Lateral flow dipstick (LFD)	BHI inoculated	30 min LAMP	2.82 CFU mL^{-1}	[17]
	hly			40 min LAMP	2.82 x 10^3 CFU mL^{-1}	
LAMP with aptamer-conjugate d magnetic capture	*actA*	Fluorescence	Phosphate buffer saline (PBS)	3 h (45 min binding; 1 h DNA extraction; 40 min LAMP)	50 CFU mL^{-1} (LAMP); 5 CFU mL^{-1} (LAMP+aptamers)	[14]
qLAMP	*hlyA*	Fluorescence	Tris-EDTA buffer	50 min LAMP	4.5 x 10^3 BCE (Bacterial cell equivalents)	[75]
LAMP	*lmo0460*	Colorimetric	Tris-EDTA buffer	1.5 h min (60 min LAMP)	47.5 x 10^3 CFU mL^{-1}	[76]
LAMP	*plcB*	Colorimetric	BHI inoculated	70 min (60 min LAMP)	2.82 CFU mL^{-1}	[57]
LAMP	*lmo0753*	Electrophoresis	PBS	3 h (60 min LAMP)	38 CFU mL^{-1}	[77]
LAMP	*actA*	Impedance	n.d.	45 min LAMP	10 copies	[78]
LAMP	*plcB*	Quartz Crystal Microbalance (QCM)	Brain heart infusion (BHI) inoculated	70-90 min (60 min LAMP)	0.3-3 CFU mL^{-1}	[79]
LAMP	*lmo0460*	Colorimetric/Electrochemical	TE buffer	2 h (20 min LAMP)	6.8 CFU mL^{-1}	[58]
On-chip LAMP	*actA*	Fluorescence	BHI inoculated	35 min LAMP	4.17 x 10^{-1} pg mL^{-1}	[80]
cLAMP	*ssrA*	Colorimetric	PBS	1 h (50 min LAMP; 5 min color development)	1 CFU mL^{-1}	[15]
PMA-LAMP	*hlyA*	Nanozyme strip	Tris buffer pH 8	70 min (LAMP)	10 CFU mL^{-1}	[81]
LAMP	*prfA*	Electrophoresis	PBS	55 min LAMP	0.5 CFU mL^{-1} (1.98 fg μL^{-1})	Present study

S9- Evaluation of P100-MP mediated lysis of *L. monocytogenes* isolated from pasteurized milk and pure culture

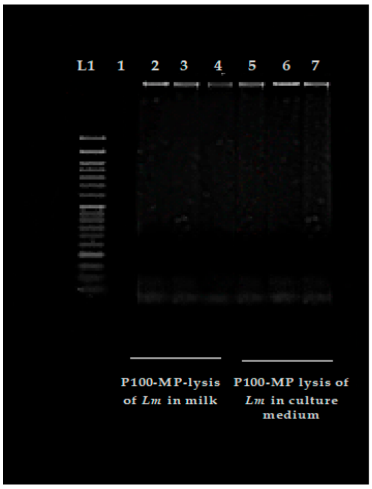

Figure S9. Evaluation of P100-MBs mediated lysis of *L. monocytogenes* isolated from pasteurized milk and pure culture. Lanes: L1 – DNA ladder; 1 – *L. monocytogenes*-blank-MBs (negative control-P100 devoid, 10^3 CFU mL^{-1}); 2-7 – P100-MBs mediated lysis of *L. monocytogenes*: 10^2 CFU mL^{-1} (2,5), 10 CFU mL^{-1} (3,6), 5 CFU mL^{-1} (4,7).

S10- Results of probit analysis in milk samples - LOD_{95}

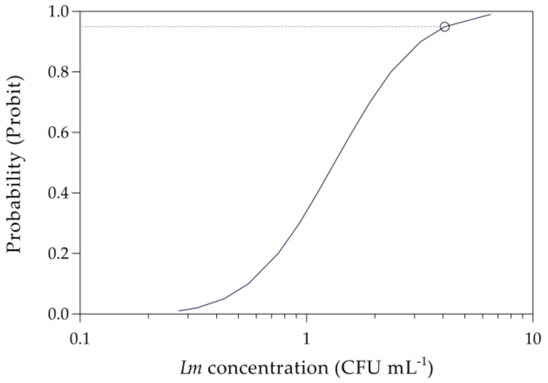

Probability	95% Confidence Limits		
	Estimate	Lower Bound	Upper Bound
0.01	0.27	0.02	0.49
0.05	0.43	0.06	0.65
0.1	0.56	0.13	0.77
0.2	0.75	0.32	0.96
0.3	0.93	0.58	1.20
0.4	1.12	0.84	1.65
0.5	1.33	1.05	2.52
0.6	1.58	1.22	4.11
0.7	1.90	1.40	7.14
0.8	2.36	1.62	13.82
0.9	3.19	1.96	34.91
0.95	4.08	2.29	75.36
0.99	6.50	3.04	320.80

S11- Endpoint electrochemical detection of LAMP amplicons

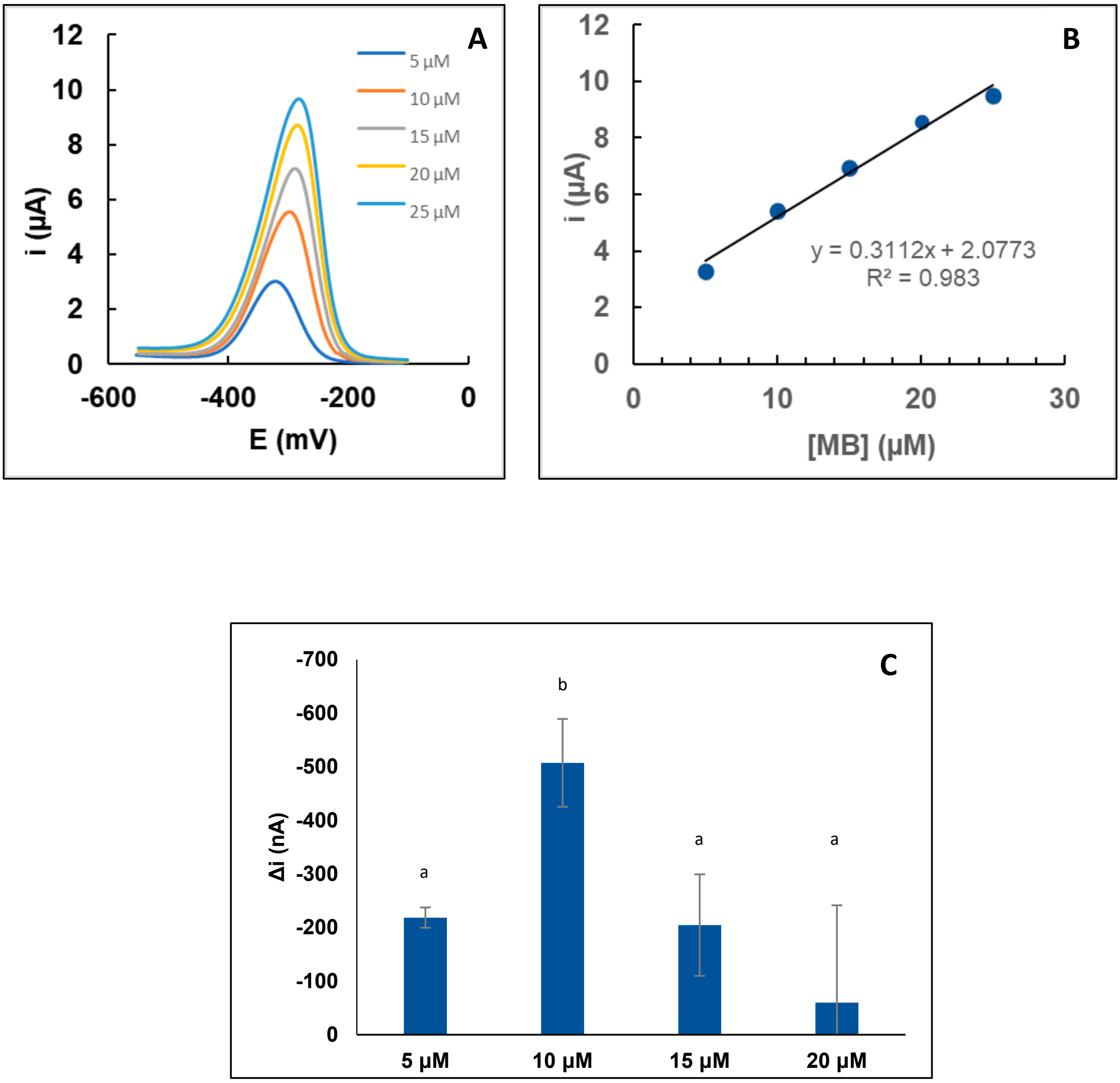

Figure S11. (A) Peak currents of MBlue at the indicated concentrations (5–25 mM) obtained in 0.01M Tris pH 7.2 with 0.02M KCl; (B) Graphical representation of the average values of peak current (n = 4) *versus* MBlue concentration; (C) Peak current variation (average values with standard deviation (n = 4)) for different concentrations of MBlue solution upon mixing 1:1 with LAMP amplified DNA (10^5 CFU mL^{-1}). Different lowercase letters indicate statistical significance ($p < 0.05$). The square wave scans were obtained with 0.025 V amplitude, 0.004 V step potential and 100 Hz frequency.

S12- Analysis of the literature on LAMP detection of *L. monocytogenes* in milk

LAMP format	Target gene	Detection approach	Test matrix	Enrichment	Detection time	LOD	Reference
LAMP	*iap*	Fluorescence	Raw milk	n.a.	43 min (LAMP)	186 CFU mL^{-1}	[69]
LAMP-chemosensor	*iap*	Colorimetric	Raw milk	n.a.	30 min (10 min LAMP)	186 CFU mL^{-1}	[71]
LAMP	*prfA*	Fluorescence	n.d.	24 h in LEB	19.5 min (LAMP)	2.22 CFU mL^{-1}	[48]
Multiple inner primers-LAMP	*hlyA*	Fluorescence	n.d.	n.a.	35 min (LAMP)	2.4 x 10^4 CFU mL^{-1}	[82]
LAMP	*hlyA*	Fluorescence	Pasteurized whole and skim milk	48 h in Half Fraser Broth	45 min (30 min LAMP)	8 CFU g^{-1}	[83]
CAMP	*hlyA*	Fluorescence	Pasteurized milk	16 h in Luria-Bertani broth	45 min (LAMP)	1 CFU mL^{-1}	[68]
LAMP-on-a-chip	*hly*	Surface-enhanced Raman scattering (SERS)	UHT milk	24 h in Modified TA10 broth	60 min (LAMP)	3.6 x 10^2 CFU mL^{-1}	[70]
Multiplex LAMP with immune-magnetic separation	*hly*	Fluorescence	UHT milk, infant formula and naturally spoiled milk	24 h in ONE Broth-Listeria	80 min (20 min cleaning; 18 min concentration; 12 min DNA extraction; 30 min LAMP)	0.11 CFU g^{-1}	[20]
LAMP	*plcA*	Colorimetric	UHT, fresh and raw milk	7 h in Tryptic Soy Broth	9 h (1 h LAMP)	3.16 CFU mL^{-1} (UHT 2.08; Fresh 3.26; Raw 5.2)	[84]
LAMP with phagomagnetic separation	*prfA*	Electrochemical	Pasteurized whole milk	n.a.	2.5 h (55 min LAMP)	1 CFU mL^{-1}	Present study

References

14. Feng, J.; Dai, Z.; Tian, X.; Jiang, X. Detection of *Listeria monocytogenes* Based on Combined Aptamers Magnetic Capture and Loop-Mediated Isothermal Amplification. *Food Control* **2018**, *85*, 443–452, doi:10.1016/j.foodcont.2017.10.027.

15. Lee, J.E.; Kim, S.A.; Mun, H.; Kim, S.R.; Ha, K.S.; Shim, W.B. A Rapid and Colorimetric Loop-Mediated Isothermal Amplification (LAMP) Based on HRP-Mimicking Molecular Beacon for the Detection of Major 6 *Listeria* Species in Enoki Mushroom. *Food Control* **2022**, *133*, 108569, doi:10.1016/j.foodcont.2021.108569.

17. Wachiralurpan, S.; Sriyapai, T.; Areekit, S.; Kaewphinit, T.; Sriyapai, P.; Santiwatanakul, S.; Chansiri, K. Development of a Rapid Screening Test for *Listeria monocytogenes* in Raw Chicken Meat Using

Loop-Mediated Isothermal Amplification (LAMP) and Lateral Flow Dipstick (LFD). *Food Anal. Methods* **2017**, *10*, 3763–3772, doi:10.1007/s12161-017-0949-4.

20. Roumani, F.; Azinheiro, S.; Carvalho, J.; Prado, M.; Garrido-Maestu, A. Loop-Mediated Isothermal Amplification Combined with Immunomagnetic Separation and Propidium Monoazide for the Specific Detection of Viable *Listeria monocytogenes* in Milk Products, with an Internal Amplification Control. *Food Control* **2021**, *125*, 107975, doi:10.1016/j.foodcont.2021.107975.

29. Simon, M.C.; Gray, D.I.; Cook, N. DNA Extraction and PCR Methods for the Detection of *Listeria monocytogenes* in Cold-Smoked Salmon. *Appl. Environ. Microbiol.* **1996**, *62*, 822–824. https://doi.org/10.1128/aem.62.3.822-824.1996.

48. Cho, A.-R.R.; Dong, H.-J.J.; Seo, K.-H.H.; Cho, S. Development of a Loop-Mediated Isothermal Amplification Assay for Detecting *Listeria monocytogenes PrfA* in Milk. *Food Sci. Biotechnol.* **2014**, *23*, 467–474, doi:10.1007/s10068-014-0064-x.

56. Wachiralurpan, S.; Sriyapai, T.; Areekit, S.; Sriyapai, P.; Thongphueak, D.; Santiwatanakul, S.; Chansiri, K. A One-Step Rapid Screening Test of *Listeria monocytogenes* in Food Samples Using a Real-Time Loop-Mediated Isothermal Amplification Turbidity Assay. *Anal. Methods* **2017**, *9*, 6403–6410, doi:10.1039/C7AY01750B.

57. Wachiralurpan, S.; Sriyapai, T.; Areekit, S.; Sriyapai, P.; Augkarawaritsawong, S.; Santiwatanakul, S.; Chansiri, K. Rapid Colorimetric Assay for Detection of *Listeria monocytogenes* in Food Samples Using LAMP Formation of DNA Concatemers and Gold Nanoparticle-DNA Probe Complex. *Front. Chem.* **2018**, *6*, 1–9, doi:10.3389/fchem.2018.00090.

58. Chen, Q.; Yao, C.; Yang, C.; Liu, Z.; Wan, S. Development of an *In-Situ* Signal Amplified Electrochemical Assay for Detection of *Listeria monocytogenes* with Label-Free Strategy. *Food Chem.* **2021**, *358*, 129894, doi:10.1016/j.foodchem.2021.129894.

59. Wang, L.; Li, Y.; Chu, J.; Xu, Z.; Zhong, Q. Development and Application of a Simple Loop-Mediated Isothermal Amplification Method on Rapid Detection of *Listeria monocytogenes* Strains. *Mol. Biol. Rep.* **2012**, *39*, 445–449, doi:10.1007/s11033-011-0757-7.

68. Li, W.; Mao, R.; Yue, X.; Wu, J.; Wu, R.; Qiao, Y.; Peng, Q.; Shi, B.; Luo, Y.; Chen, X.; et al. Competitive Annealing Mediated Isothermal Amplification (CAMP) for Rapid and Simple Detection of *Listeria monocytogenes* in Milk. *Food Control* **2020**, *117*, 107347, doi:10.1016/j.foodcont.2020.107347.

69. Wang, D.; Huo, G.; Ren, D.; Li, Y. Development and Evaluation of a Loop-Mediated Isothermal Amplification (LAMP) Method for Detecting *Listeria monocytogenes* in Raw Milk. *J. Food Saf.* **2010**, *30*, 251–262, doi:10.1111/j.1745-4565.2009.00196.x.

70. Teixeira, A.; Paris, J.L.; Roumani, F.; Diéguez, L.; Prado, M.; Espiña, B.; Abalde-Cela, S.; Garrido-Maestu, A.; Rodriguez-Lorenzo, L. Multifuntional Gold Nanoparticles for the SERS Detection of Pathogens Combined with a LAMP-in-Microdroplets Approach. *Materials (Basel).* **2020**, *13*, doi:10.3390/MA13081934.

71. Wang, D.; Zhang, G.; Lu, C.; Deng, R.; Zhi, A.; Guo, J.; Zhao, D.; Xu, Z. Rapid Detection of *Listeria monocytogenes* in Raw Milk with Loop-Mediated Isothermal Amplification and Chemosensor. *J. Food Sci.* **2011**, *76*, doi:10.1111/j.1750-3841.2011.02383.x.

72. Tang, M.J.; Zhou, S.; Zhang, X.Y.; Pu, J.H.; Ge, Q.L.; Tang, X.J.; Gao, Y.S. Rapid and Sensitive Detection of *Listeria monocytogenes* by Loop-Mediated Isothermal Amplification. *Curr. Microbiol.* **2011**, *63*, 511–516, doi:10.1007/s00284-011-0013-3.

73. Wan, C.; Yang, Y.; Xu, H.; Aguilar, Z.P.; Liu, C.; Lai, W.; Xiong, Y.; Xu, F.; Wei, H. Development of a Propidium Monoazide Treatment Combined with Loop-Mediated Isothermal Amplification (PMA-LAMP) Assay for Rapid Detection of Viable *Listeria monocytogenes*. *Int. J. Food Sci. Technol.* **2012**, *47*,

2460–2467, doi:10.1111/j.1365-2621.2012.03123.x.

74. Pisamayarom, K.; Suriyasomboon, A.; Chaumpluk, P. Simple Screening of *Listeria monocytogenes* Based on a Fluorescence Assay via a Laminated Lab-on-Paper Chip. *Biosensors* **2017**, *7*, doi:10.3390/bios7040056.

75. Garrido-Maestu, A.; Azinheiro, S.; Carvalho, J.; Fuciños, P.; Prado, M. Development and Evaluation of Loop-Mediated Isothermal Amplification, and Recombinase Polymerase Amplification Methodologies, for the Detection of *Listeria monocytogenes* in Ready-to-Eat Food Samples. *Food Control* **2018**, *86*, 27–34, doi:10.1016/j.foodcont.2017.11.006.

76. Liu, Z.; Yao, C.; Wang, Y.; Yang, C. A G-Quadruplex DNAzyme-Based LAMP Biosensing Platform for a Novel Colorimetric Detection of *Listeria monocytogenes*. *Anal. Methods* **2018**, *10*, 848–854, doi:10.1039/c7ay02908j.

77. Nathaniel, B.R.; Ghai, M.; Druce, M.; Maharaj, I.; Olaniran, A.O. Development of a Loop-Mediated Isothermal Amplification Assay Targeting Lmo0753 Gene for Detection of *Listeria monocytogenes* in Wastewater. *Lett. Appl. Microbiol.* **2019**, *69*, 264–270, doi:10.1111/lam.13200.

78. Sharif, S.; Wang, Y.; Ye, Z.; Wang, Z.; Qiu, Q.; Ying, S.; Ying, Y. A Novel Impedimetric Sensor for Detecting LAMP Amplicons of Pathogenic DNA Based on Magnetic Separation. *Sensors Actuators, B Chem.* **2019**, *301*, 127051, doi:10.1016/j.snb.2019.127051.

79. Wachiralurpan, S.; Chansiri, K.; Lieberzeit, P.A. Direct Detection of *Listeria monocytogenes* DNA Amplification Products with Quartz Crystal Microbalances at Elevated Temperatures. *Sensors Actuators, B Chem.* **2020**, *308*, 127678, doi:10.1016/j.snb.2020.127678.

80. Jin, J.; Duan, L.; Fu, J.; Chai, F.; Zhou, Q.; Wang, Y.; Shao, X.; Wang, L.; Yan, M.; Su, X.; et al. A Real-Time LAMP-Based Dual-Sample Microfluidic Chip for Rapid and Simultaneous Detection of Multiple Waterborne Pathogenic Bacteria from Coastal Waters. *Anal. Methods* **2021**, *13*, 2710–2721, doi:10.1039/d1ay00492a.

81. Shi, D.; Shi, H. Combining Loop-Mediated Isothermal Amplification and Nanozyme-Strip for Ultrasensitive and Rapid Detection of Viable *Listeria monocytogenes* Cells and Biofilms. *LWT - Food Sci. Technol.* **2022**, *154*, 112641, doi:10.1016/j.lwt.2021.112641.

82. Wang, Y.; Wang, Y.; Ma, A.; Li, D.; Luo, L.; Liu, D.; Hu, S.; Jin, D.; Liu, K.; Ye, C.; et al. The Novel Multiple Inner Primers-Loop-Mediated Isothermal Amplification (MIP-LAMP) for Rapid Detection and Differentiation of *Listeria monocytogenes*. *Molecules* **2015**, *20*, 21515–21531, doi:10.3390/molecules201219787.

83. Tirloni, E.; Bernardi, C.; Drago, S.; Stampone, G.; Pomilio, F.; Cattaneo, P.; Stella, S. Evaluation of a Loop-Mediated Isothermal Amplification Method for the Detection of *Listeria monocytogenes* in Dairy Food. *Ital. J. Food Saf.* **2017**, *6*, 179–184, doi:10.4081/ijfs.2017.6890.

84. Azinheiro, S.; Roumani, F.; Prado, M.; Garrido-Maestu, A. Rapid Same-Day Detection of *Listeria monocytogenes*, *Salmonella* spp., and *Escherichia coli* O157 by Colorimetric LAMP in Dairy Products. *Food Anal. Methods* **2022**, 2959–2971, doi:10.1007/s12161-022-02345-9.
